# Open habitats and species differences shape space use in semi-feral cattle and horses across Danish rewilding sites

**DOI:** 10.1007/s10661-026-15487-8

**Published:** 2026-05-23

**Authors:** Marianne Damholdt Bergin, Luca Börger, Érika Garcez da Rocha, Rasmus Worsøe Havmøller, Mads Jensen, Jens-Christian Svenning

**Affiliations:** 1 Center for Ecological Dynamics in a Novel Biosphere (ECONOVO), Department of Biology, Aarhus University, Ny Munkegade 114, DK- 8000 Aarhus C, Denmark; 2 Danish Nature Agency, Førstballevej 2, 7183 Randbøldal, Denmark; 3 Department of Biosciences, Swansea University, Swansea, Wales UK; 4 Department of Biology, University of Saskatchewan, Saskatoon, SK Canada; 5 Department of Zoology, Natural History Museum Denmark, University of Copenhagen, Copenhagen, OE Denmark; 6 Natural History Museum and Botanical Garden, University of Agder, Kristiansand, Norway

**Keywords:** Trophic rewilding, Habitat use, Resource selection functions, Large herbivores, Semi-feral cattle, Semi-feral horses, Foraging ecology

## Abstract

Large herbivores can strongly influence ecosystem structure and biodiversity, making knowledge of their space use important for trophic rewilding. We used GPS-tracking data collected over two years from 10 semi-feral cattle and 17 horses across ten Danish rewilding sites to analyse habitat use in a use–availability framework using resource selection functions (RSFs), complemented by direct behavioural observations. Both species showed large individual variability but overall used habitats disproportionately to availability, with higher relative use of open habitats; including dry grassland and heath–dune habitats, and lower relative use of dense shrub, conifer forest, mixed and broadleaved forest and wetlands relative to grasslands. Horses showed higher relative use of open wet and heath–dune habitats compared with cattle. Behavioural observations supported marked interspecific differences in activity budgets, with horses spending most observed time grazing and cattle allocating more time to resting and ruminating. Habitat use also varied seasonally, including increased use of wetlands and forests in summer, while supplementary winter feeding caused strong local shifts in habitat use by concentrating animal activity around winter feeding areas. These results show that habitat composition and management strongly determine where herbivore impacts are likely to be concentrated within rewilding sites, and that dense conifer-dominated stands are likely to receive relatively limited use by horses and cattle. Thus, considering herbivore space-use ecology in site planning and management and introducing herbivores with distinct digestive physiologies and foraging strategies such as horses and cattle, will improve the effectiveness of trophic rewilding.

**Supplementary Information:**

The online version contains supplementary material available at 10.1007/s10661-026-15487-8.

## Introduction

There is a growing interest and increased investment in rewilding worldwide (Jepson & Schepers, 2016) including the trophic rewilding approach, which aims to restore ecological processes through the recovery of missing trophic interactions, especially those involving large herbivores and carnivores (Svenning et al., 2016, 2024). In practice, however, trophic rewilding commonly involves the introduction or re-establishment of large herbivores in fauna-depleted, human-modified landscapes (Child et al., 2019; Pedersen et al., 2020) with the aim of promoting more self-regulating and ecologically dynamic ecosystems (Bakker & Svenning, 2018; Hughes et al., 2011; Perino et al., 2019; Svenning, 2020). These practices are increasingly reflected in EU policy frameworks promoting ecosystem restoration, including the European biodiversity Strategy 2030 and the EU Nature Restoration Regulation (European Commission, 2020, 2024).

Across Europe, implementation of trophic rewilding often takes place in fenced and managed settings that differ markedly in habitat composition, vegetation structure, and land-use history. In such systems, evaluating rewilding outcomes requires not only knowledge of which herbivores are present, but also of how they use the landscapes into which they are introduced. Herbivore space use is central to this issue because it shapes where ecological processes are expressed within sites (Schlägel et al., 2020). Through their movements and uneven use of habitats, large herbivores influence the spatial distribution of grazing (Tews et al., 2004) trampling, resting, nutrient redistribution, and seed dispersal (Kastovska et al., 2024; Schmitz et al., 2023), thereby contributing to vegetation heterogeneity and broader ecosystem dynamics (Krause et al., 2025). Empirical knowledge of habitat use is therefore important not only for interpreting rewilding outcomes, but also for assessing and predicting where herbivore impacts are likely to be concentrated within managed rewilding sites (Gomez et al., 2025). This is particularly relevant in monitoring contexts (Allen & Singh, 2016), where habitat use data can help link animal presence and movement patterns to the spatial distribution of ecological effects (Tews et al., 2004).

Trophic rewilding and conservation management in Europe often include introducing semi-feral horses and cattle to promote ecological processes (Putfarken et al., 2008). Equids and bovids are generalist herbivores of similar body size that co-exist in many natural and semi-natural ecosystems (Menard et al., 2002). Yet, they differ fundamentally in digestive physiology and foraging strategy. Horses are grazers and hindgut fermenters, characterised by rapid gut passage rates and relatively low digestive efficiency, which requires them to consume large quantities of forage and forage more continuously to meet energetic demands (Forbes & Kerley, 2022; Schoenecker et al., 2016). As a result, horses are often able to exploit abundant but often lower-quality, fiber-rich grasses and are expected to spend a large proportion of time actively feeding in open habitats where forage intake rates are maximised. In contrast, cattle are ruminant foregut fermenters, with high digestive efficiency due to extensive microbial fermentation in the rumen, allowing them to extract energy effectively from fibrous plant material, but at the cost of slower digestion and the need for prolonged rumination (Hofmann, 1989; Van Soest, 1994). These fundamental physiological and behavioural differences provide a mechanistic basis for expecting species-specific patterns of habitat and space use across heterogeneous landscapes, with implications for how horses and cattle distribute grazing pressure and influence vegetation structure in trophic rewilding systems. Knowledge of the comparative space use and foraging behaviour of cattle and horses in restoration sites managed according to trophic rewilding principles is therefore critical for implementing successful rewilding projects (Menard et al., 2002).

Despite the practical relevance of these issues, current knowledge of horse and cattle movement patterns is of limited relevance to rewilding, as most available studies focus either on domesticated animals in agricultural systems or on wild populations inhabiting extensive wilderness landscapes (Kaczensky et al., 2010; King, 2002). Furthermore, studies of the effects of cattle and horses in conservation management are most often conducted on single species at single sites (Lovász et al., 2024; Mirski, 2021; Putfarken et al., 2008), emphasizing the importance of obtaining empirical data on habitat selection in rewilding contexts. We therefore require more knowledge across sites and herbivore species on space use behaviour to support the growing investment in trophic rewilding.

The ecological effects of trophic rewilding therefore depend not only on which large herbivores are present, but also on how they use the habitats available within a site. The degree to which a site’s rewilding potential is realized can be assessed by the extent to which suitable habitats are occupied by the large herbivores (Mata et al., 2021) and thus their potential to affect ecosystems through trophic interactions (Svenning, 2020; Svenning et al., 2016). Knowledge of the comparative habitat use and foraging behaviour of cattle and horses under wilder conservation management schemes based on trophic rewilding principles is therefore required to support successful rewilding implementation (Menard et al., 2002). Such knowledge can aid practitioners and planners in site selection and management, enable appropriate monitoring of ecological effects, and facilitate assessment of the spatial progression of rewilding sites (Mata et al., 2021).

Here, we investigate how semi-feral cattle and horses use available habitats across ten Danish rewilding sites and whether their habitat use differs in ways consistent with their contrasting digestive physiology and foraging strategies. We also assess how habitat use varies seasonally and how it is influenced by management interventions such as supplementary winter feeding, and consider what these patterns imply for the short-term spatial distribution of herbivore activity within rewilding sites.

## Materials and methods

### Study areas

**Fig. 1:**
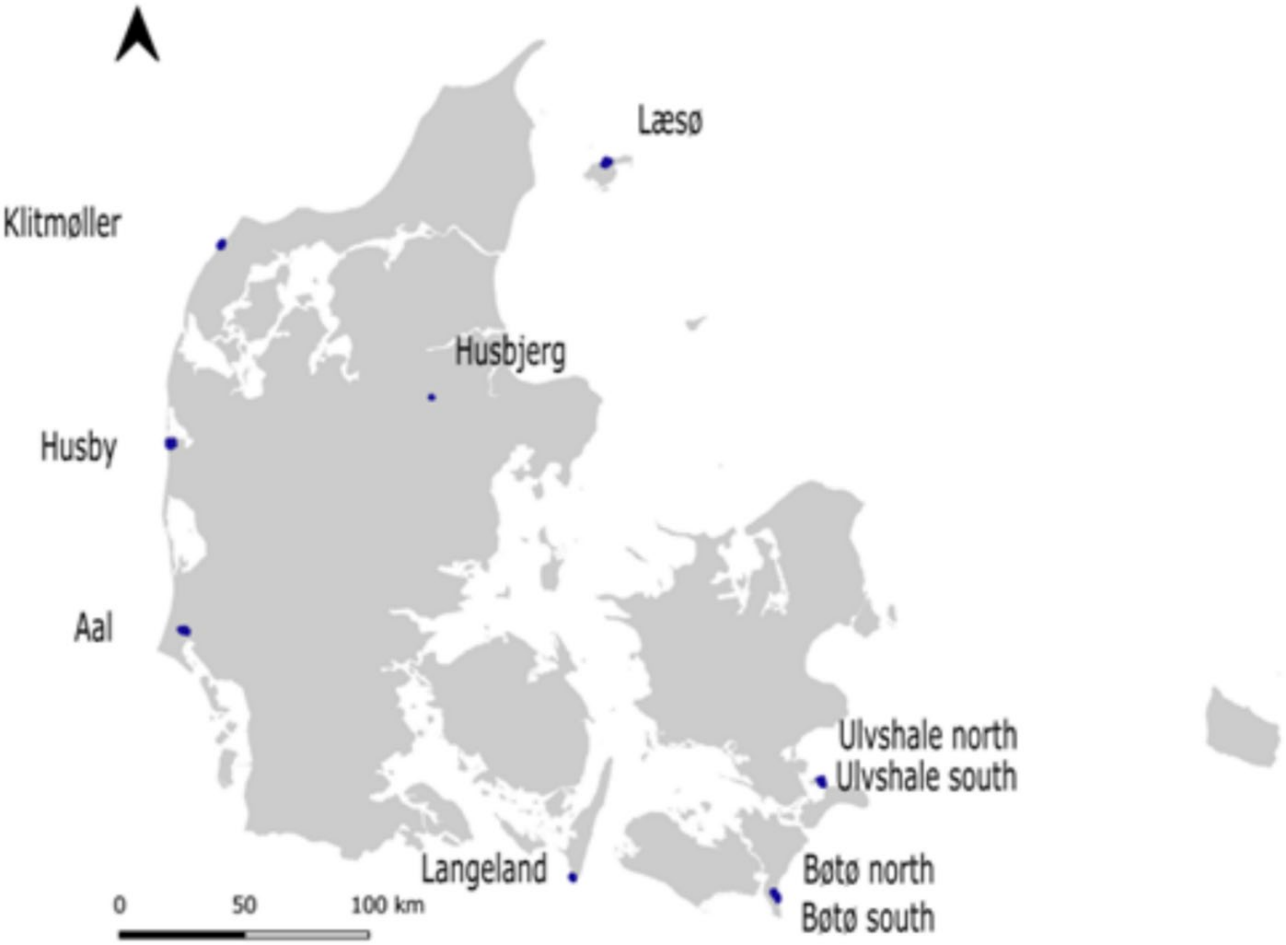
Location of the ten study sites across Denmark for the study of semi-feral horse and cattle habitat selection. GPS-location data was collected from 2021 to 2023 using GPS-equipment on 10 semi-feral cattle and 17 semi-feral horses

This study included 10 rewilding sites across Denmark, in the temperate European region (Fig. 1), with cattle and horses in year-round extensive grazing management (Table 1). Supplementary feeding was generally not provided, however, hay was provided during the winter months at five sites — Langeland, Aal, Læsø, Husbjerg and Klitmøller. Cattle and horses co-occurred at three sites: Husby, Langeland and Ulvshale South. The management of the sites included in this study ranged from “minimal rewilding” to “partial rewilding”, according to the TRAAIL framework (Pedersen et al., 2020) while the wildness state was assessed to range from “intensively managed” to “simulated natural”, according to the framework of Child et al. (2019). This reflects the ongoing population management with a low occurrence of natural social breeding herds and fences restricting movements, resulting in less than natural or wild population dynamics and movement patterns. The study sites were located in regions with very limited or no presence of large predators such as wolves, and where predation risk for adult horses and cattle was generally negligible.

**Table 1:** Overview of study areas and GPS tracking; location, herd size, area size, type of animal tagged with GPS trackers (sex and race), number of tagged animals (cattle *n* = 10, horses *n* = 17), the equipment used and study periods

Group	Location	Herd size (max)	Area size (ha)	Race	Tagged animal type	Tracked animals	Equipment	Period of data included
Cattle	Aal	23	362	Galloway	Bullocks	2	Digitanimal collar	May 2022–Nov 2023
	Læsø	26	359	Galloway	Bullocks	2	Digitanimal collar	June 2022–June 2023
	Husbjerg	8	22.5	Mix	Bullocks	1	Digitanimal collar	Nov 2022–Nov 2023
	Husby	8	381	Highland	Bullocks	2	Digitanimal collar	Aug 2022–Nov 2023
	Langeland	7	107	Highland	Stud	1	Digitanimal collar Anicare ear tag	Nov 2021–Nov 2023
	Ulvshale South	32	139	Galloway	Bullocks	2	Digitanimal collar Anicare ear tag	Aug 2022–Nov 2023
Horse	Bøtø south	24	148	Konik	Stallion	1	Vectronic Vertex Plus	Jan 2022–Jan 2023
	Bøtø north	17	193	Konik	Stallion, geldings	1	Vectronic Vertex Plus	Jan 2022–Aug 2023
	Langeland	45	107	Exmoor	Mares	9	Digitanimal collar	Nov 2021–Nov 2023
	Husby	17	381	Exmoor/Shetland	Geldings	2	Digitanimal collar,	Aug 2022–Nov 2023
	Klitmøller	10	228	Konik	Geldings	2	Digitanimal collar	Aug 2022–Nov 2023
	Ulvshale North	6	105	Exmoor	Geldings, mares	1	Digitanimal collar	Aug 2022–Nov 2023
	Ulvshale South	8	139	Exmoor	Geldings, mares	1	Digitanimal collar	Aug 2022–Nov 2023

Sites consist of diverse vegetation and variation in cover, from predominantly open sites to sites with large areas of woodland cover. We mapped habitats into eight discrete categorical habitat types based on existing maps of protected habitat types (Danish Environmental Portal,  n.d.) orthophotos and ground truthing. We simplistically ranked habitats’ expected average available forage and productivity along a scale from open to densely and shaded vegetated habitat types inspired by Buttenschøn (2007); Ebrahimi et al. (2010); however, we note that productivity is not static, but strongly influenced by spatio-temporally dynamics with seasonal variation in forage yield, crude protein, and digestible energy across habitat types (Supplementary information Table S1).

### Study animals

We deployed global positioning system (GPS) trackers on adult semi-feral cattle and horses. The sampling period spanned two years from October 2021 to October 2023, during which a total of 10 cattle and 17 horses at 10 different sites were fitted with GPS trackers (Table 1). For each animal, the sampling period was at least one year. GPS trackers included GPS-collars from Digitanimal (Digitanimal S.L.) and Vectronic Vertex Plus (Vectronic Aerospace Gmbh); two cattle were additionally tagged with Rudolf GPS ear tags (Anicare Oy) (GPS-tag average accuracy was 25 m (Pedersen & Havmøller, 2025)). Data transmission methods included GSM, Sigfox and NB-IoT. GPS sampling frequency varied between trackers, from 30 to 60 min sampling intervals.

### Data management

All location data was mapped and visually inspected and we removed outliers located more than 20 m outside fence lines. Locations within 20 m of the fence line were snapped to the nearest point inside the fence. For each individual, the location data was resampled to only include the first fix recorded for each hour resulting in a total 112.239 data points (see Supplementary Information Table S2 for data points per animal and Table S3 for points per season).

Both cattle and horses displayed herd behaviour, where all individuals of each species moved together in a site. Consequently, we used the tagged individuals as proxy for the herd’s movement in general, with tagged individuals representing on average 13% of the total herd size (range 4–25%, see Table 1).

### Statistical analysis

#### Resource selection function

We performed resource selection analyses, more generally also called habitat selection analysis (Fieberg et al., 2021) which provides a statistical framework to link animal locations to environmental covariates (Manly et al., 2002). We sampled 20 random locations for every used location per animal within a site using the amt package in R (version 0.2.2.0) (Signer et al., 2019). We checked that this provided sufficient available points to ensure parameter estimates converged to stable values, see Fieberg et al. (2021) and we assigned a weight of 5000 to each available location and a weight of 1 to all observed locations (Fieberg et al., 2021; Northrup et al., 2022). Following Fieberg et al. (2021), we fitted logistic regression models (GLM with binomial family link) to location data, then calculated for each animal the estimated coefficients and standard deviation and standard errors. We used exponentiated habitat selection function coefficients, which provide estimates of the relative selection strength of a habitat category compared to a reference category. We used open grassland as reference category, as open grassland is the only habitat present in all study sites and is expected to be a key feeding habitat for both cattle and horses. Hence, the coefficients for categorical predictors reflect use:availability ratios for each habitat relative to the use:availability ratio for the reference habitat grassland. To correct for the unequal availability of the habitat categories within each of the sites, we multiplied the estimated coefficients by the ratio of the availability of each habitat compared to the reference habitat and furthermore converted selection coefficients into relative use (following Fieberg et al. (2021)). To obtain population-level results, we averaged the individual-level adjusted relative use coefficients across individuals for each habitat category, using a bootstrap approach (10000 replicates) to obtain robust estimates of the distribution of the estimated coefficients (Crawley, 2012).

Furthermore, to investigate temporal changes, we divided the year into seasons using the astronomical seasons based on the equinoxes, when the length of the day and night are equal, and the midpoints, the winter and summer solstices. Thus, we divided the data into the four astronomical seasons: spring (March 20–June 21), summer (June 22–September 22), autumn (September 23–December 21), and winter (December 22–March 19) and then fitted the glm model to each individual/season combination.

To test if habitat selection overall differed between seasons, we added an additional mixed effects analysis for each species, using the function glmer in the lme4 (version 1.1–34 package; (Bates et al., 2015) in R version 4.3.1 (R Core Team, 2023) with animal ID as random effect and comparing two nested models, one model included only the main effect of habitat and the other included both the main effect of habitat and the interaction between season and habitat. We used the anova() function in R to compare these nested models, using a chi-square *p*-value to assess the significance of the difference in deviance between the models, and AIC scores as a measure of support from the data model complexity.

#### Behavioural data

We performed direct observations of individual animals at nine sites (Langeland, Husby, Klitmøller, Bøtø North, Bøtø South, Læsø, Aal, Ulvshale North and Ulvshale South) during the daytime (when light allowed) and recorded behaviours, location (using handheld GPS-units) and time. Observations were carried out with 1-min intervals switching between observations of focal individuals and identifying the dominant behaviour in the herd focal sub-groups (Altmann, 1974). Behaviours were classified into 10 categories; grazing, browsing, drinking, standing, resting, walking, wallowing, lactating, rubbing, and other following the protocol by Buitenwerf and Svenning (2019) (see Supplementary Information Table S4). Observational studies were carried out in 90-min blocks, with 1-min breaks between each observation allowing for recording and this resulted in approximately 45 observations per block. However, observation blocks were shorter if animals moved out of sight.

Each recorded behaviour was assigned a habitat category corresponding to the location, according to the categorical habitat map. Observations were carried out throughout the year, however due to the relative low sample size, observational data was pooled across all sites and seasons, resulting in 808 observations for cattle and 1864 for horses. The analysis therefore does not take season or site into account.

The difference between the distribution of horse and cattle behaviours were tested using a Chi square test and the difference between cattle and horse behaviour in each habitat was tested using a Pearson’s Chi-squared test with simulated *p*-values (Monte Carlo *p*-values, 2000, due to small sample size).

## Results

### Habitat selection — evidence of avoidance and preferences

**Fig. 2:**
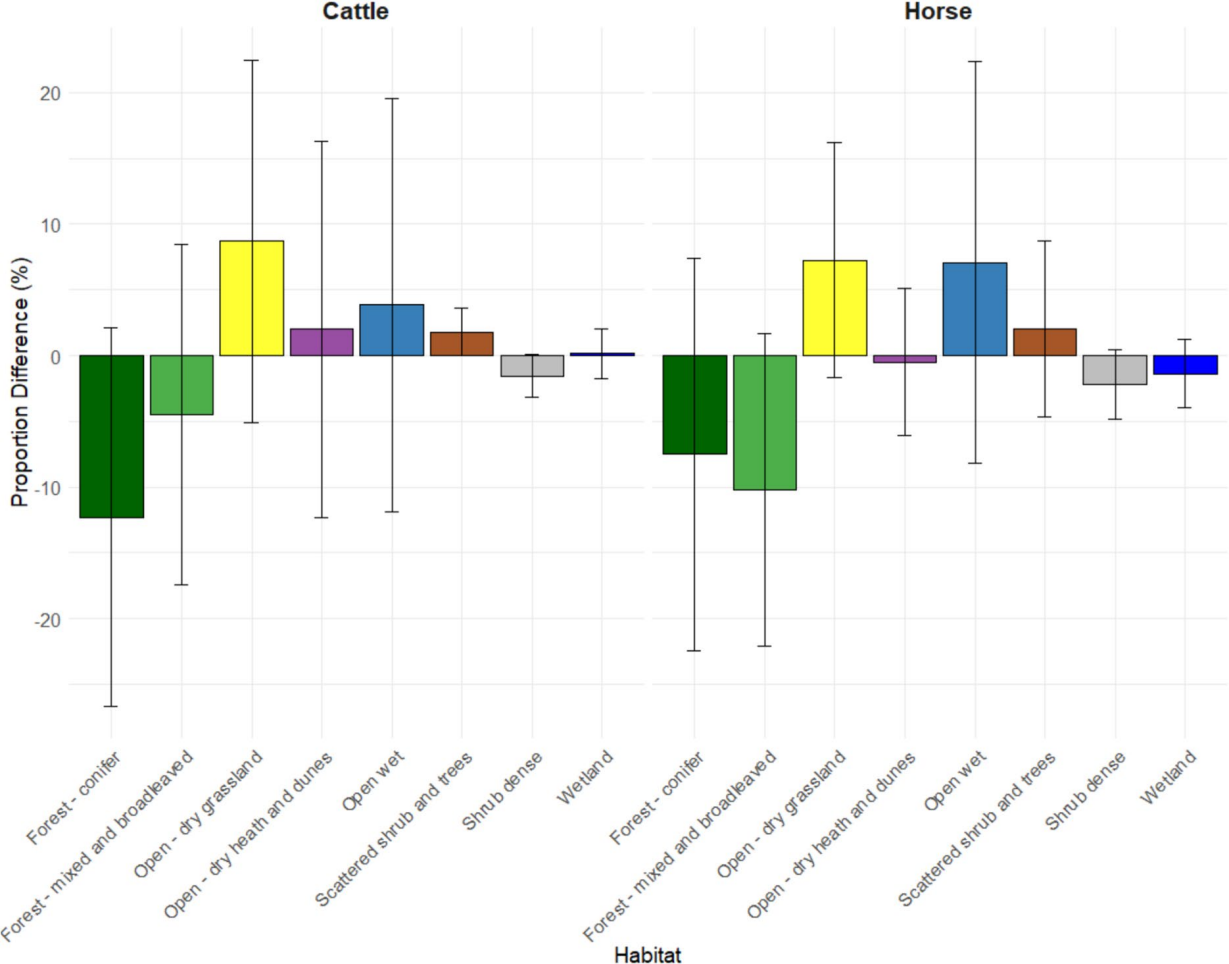
Proportional difference (%) in used and available habitat for cattle and horses across sites. Mean shown in bar with standard deviation error bars. The study was based on GPS location data on cattle (*N* = 10) and horses (*N* = 17) across ten study sites across Denmark and analysed using resource selection functions. Data spanned for up to two years, and here season is not taken into account. The use:availability ratio was generally negative for conifer and broadleaved forest, illustrating avoidance of these habitats, while the use:availability ratio of open wet and grassland habitats were generally positive, illustrating a preference for these habitats

We found strong evidence that horses and cattle did not utilise habitats equally to availability, with consistent patterns of habitat selection despite marked individual variability (Fig. 2).

**Fig. 3:**
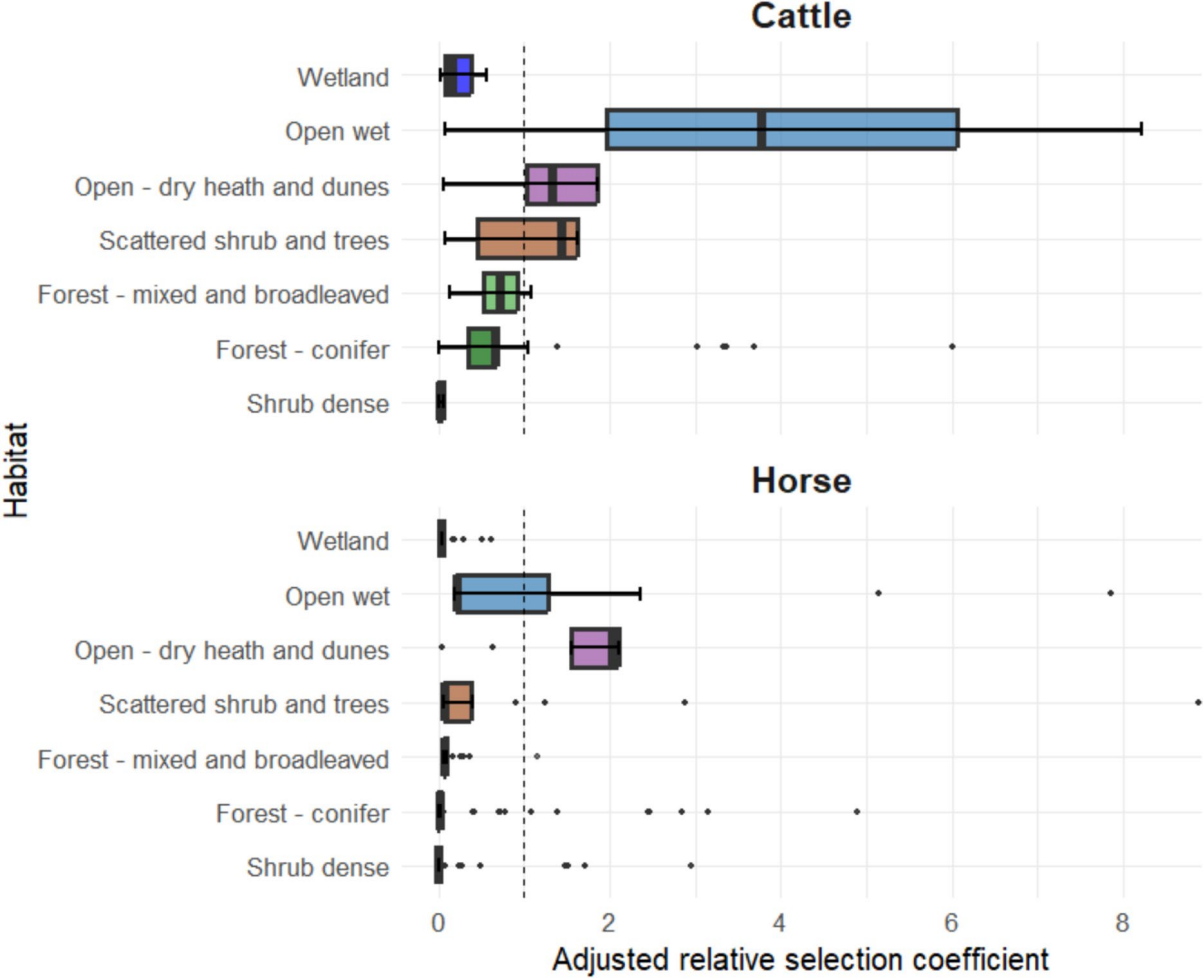
Adjusted habitat selection coefficients displayed in order of density of vegetation and expected forage availability based on resource selection function analysis. Values represent individual-level averages across animals for each habitat category, estimated using a bootstrap approach (10,000 replicates). The study was based on GPS location data from cattle (*N* = 10) and horses (*N* = 17) collected at ten study sites across Denmark. Cattle and horses co-habited at three sites: Husby, Langeland and Ulvshale South. Data spanned for up to two years, and this analysis did not take season into account. Adjusted selection coefficient of 1 represent an equal use:availability ratio to the reference category habitat, open dry grassland

Accounting for habitat availability within site, both cattle and horses showed a low relative probability of use of wetlands, dense shrub, conifer forest and mixed and broadleaved forest habitats compared to grasslands (i.e. the use:availability ratio for these habitats was less than the use:availability ratio for the reference class, grassland; see Fig. 3). In addition, there were consistent differences in habitat use between horses and cattle. Horses showed higher relative use of open-dry heath-dune habitats compared to grassland while cattle had a higher use of both open wet, open-dry heath–dune and scattered shrub habitats compared to grassland (Fig. 3) (Supplementary Information Fig. S5, Table S6, Table S7).

### Habitat use is context dependent

In sites dominated by open wet habitat and relatively little dry grassland (Husby, Bøtø North, Ulvshale South and North; see Supplementary Information Fig. S8), horses displayed a disproportionally higher use of open wet habitat relative to availability (see Supplementary Information Figs. S9 and S10). In areas dominated by open – dry heath and dune habitats with very limited grassland habitat (Klitmøller and Læsø), both horses and cattle showed higher relative use of heath and dune vegetation types. At Bøtø South, where scattered shrub and mixed woodland dominated, horses were more frequently recorded in heath-dune habitat and open dry grassland. Notably, we found horses utilised scattered shrub at levels comparable to grassland, while exhibiting lower relative use of conifer forest, mixed woodland and dense shrub. In more heavily forested sites (Aal and Husbjerg), cattle were recorded using forest habitats at levels comparable to grassland.

By converting selection coefficients into relative use, we found that in grassland-dominated Langeland, horses were 208–859 times more likely to use grassland than dense shrub and 66–408 times more likely to use grassland than conifer forest. Cattle showed a similar pattern, with grassland 319 times more likely to be used than dense shrub and 284 times more likely than conifer forest (Supplementary Information Fig. S6).

**Fig. 4:**
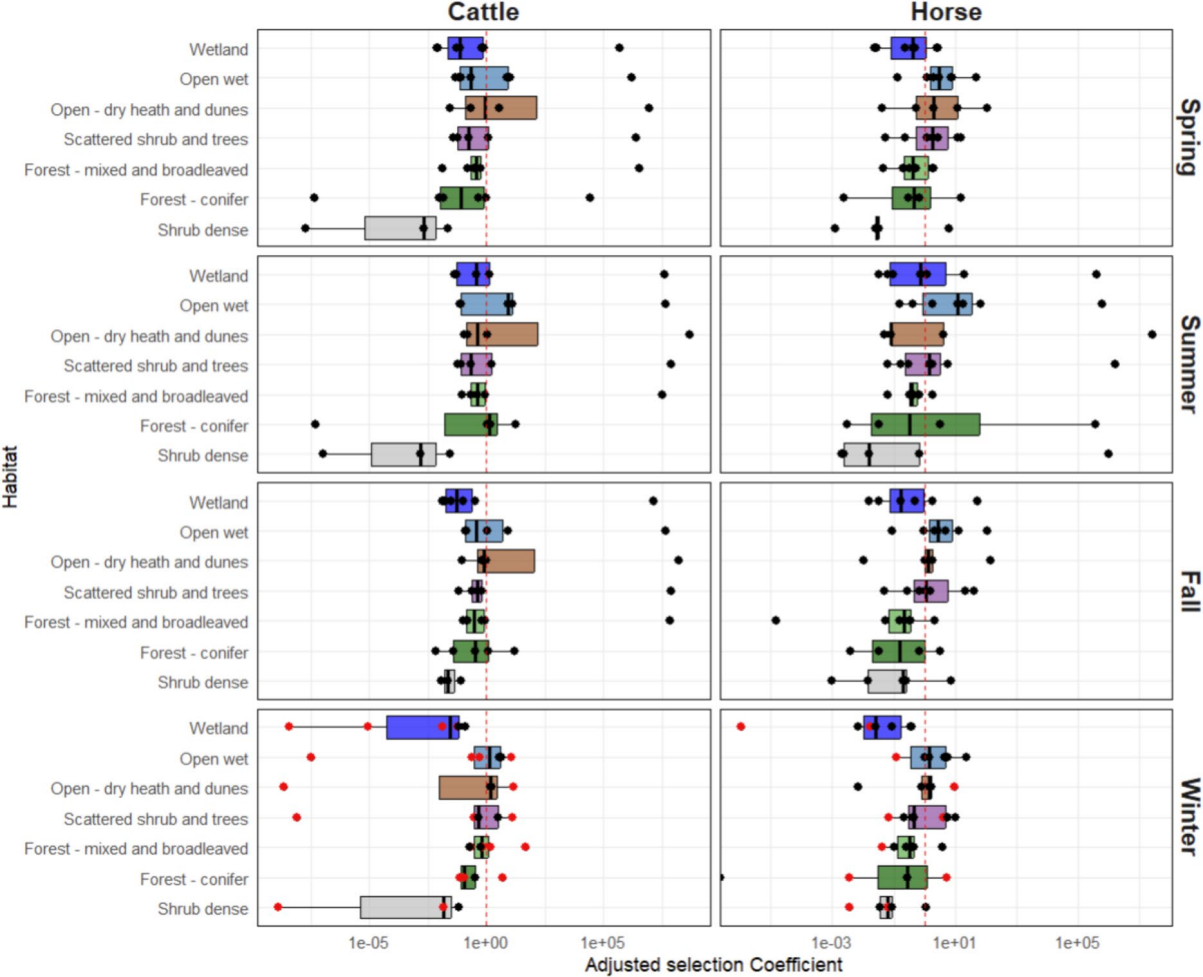
Selection coefficients, adjusted for temporal (seasons) and spatial differences (site availability), estimated with resource selection analysis, displayed in order of density of vegetation and expected forage availability (illustrated in log scale). The study was based on GPS location data from cattle (*N* = 10) and horses (*N* = 17) collected at ten study sites across Denmark, but for this illustration only one animal per site is illustrated (cattle *N* = 6, and horses *N* = 7). Cattle and horses co-occurred at three sites: Husby, Langeland and Ulvshale South

Seasonal changes in habitat use significantly differed between seasons for both cattle and horses, with the model including the interaction between habitat and season showing a significantly better fit for both cattle (*P* < 0.001) and horses (*P* < 0.001). Horses used conifer significantly more during summer, similarly wetlands were used more in the summer and avoided in winter (Fig. 4). For cattle, we observed a similar pattern of higher usage of wetlands during summer and lower relative use during winter.

In sites where hay was provided as supplementary feed during winter, we observed a marked shift in habitat use by cattle, compared to use patterns without localised resource provisioning. During winter, supplementary-fed cattle on Læsø were more frequently recorded in mixed forest habitats relative to the reference habitat (grassland), whereas in other seasons they were more often recorded in open dry heath dune habitats relative to grassland. The increased use of mixed forest coincided with the location of supplementary feeding. Similarly, during winter in Aal, cattle showed higher relative use of grassland, corresponding to the location of supplementary feeding during the winter months. For horses, we did not see a marked change in habitat use during supplementary feeding (Fig. 4).

### Behavioural differences between horses and cattle

**Fig. 5:**
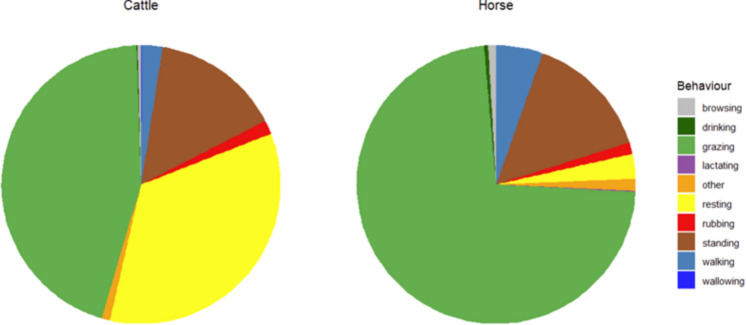
Behavioural observations of cattle (N = 808) and horses (N = 1864) recorded during daytime across nine study sites in Denmark. Horses spent the majority of observed time grazing (73%), whereas cattle grazed for 45% of the time and rested lying down (including rumination) for 35% of the time. These differences reflect the distinct digestive systems and associated foraging strategies of the two species. Both cattle and horses spent 15% of the time standing

Horses and cattle differed significantly in their observed behavioural time budget (chi-square test *P* < 0.001). Horses grazed 73% of the time observed, while cattle were observed to graze less than 50% of the time and laying resting (include rumination) 35% of the time (Fig. 5 and Supplementary Information Table S11).

**Fig. 6:**
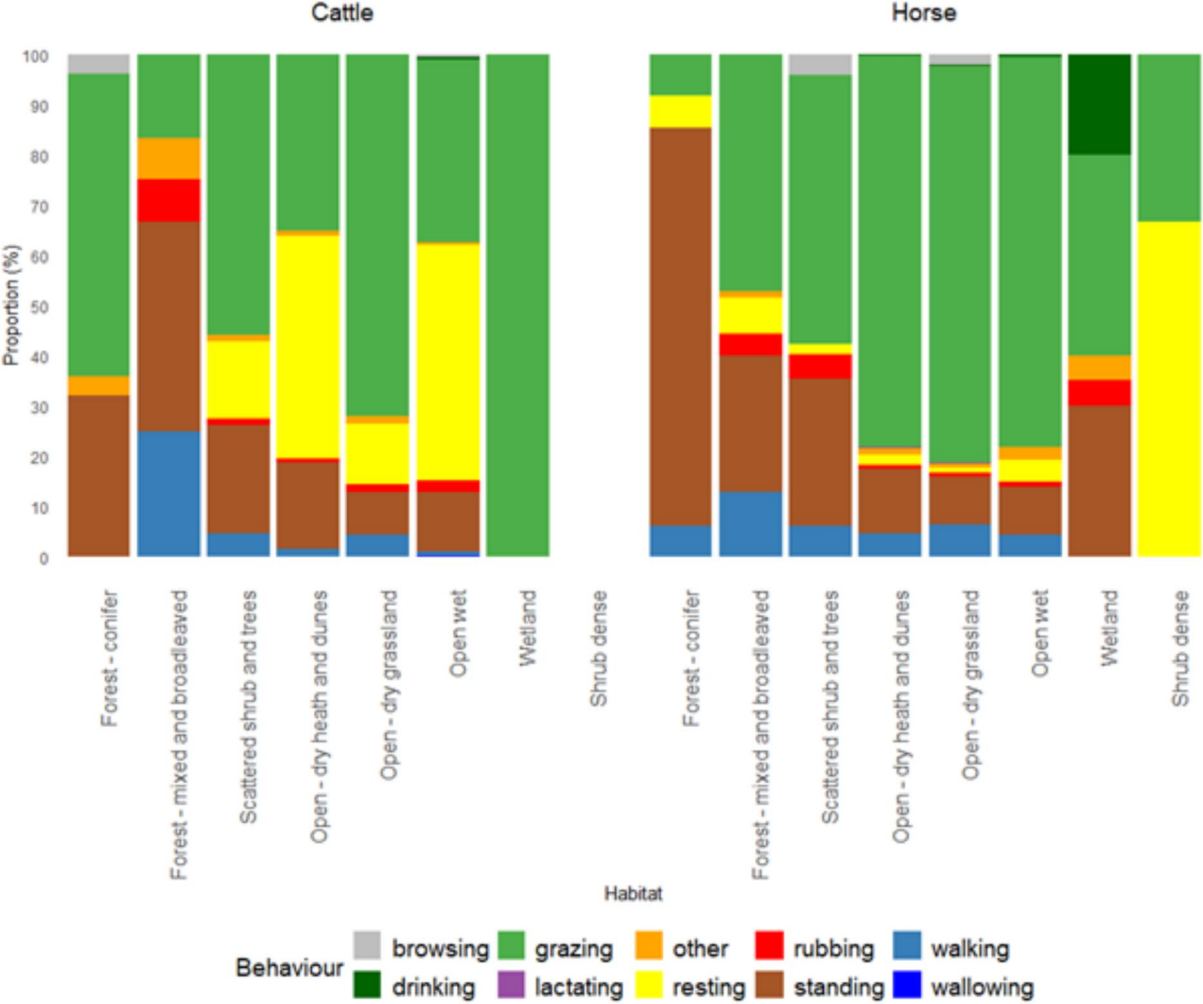
Behavioural observations of cattle (*N* = 808) and horses (*N* = 1864) across nine study sites in Denmark. Behaviour types are shown as proportional (%) to the habitat in which they occurred. Significant differences in the distribution of behaviours between species were detected in conifer forest (*P* = 0.02749), mixed and broadleaved forest (*P* = 0.02799), open dry heath and dunes (*P* = 0.02449), and scattered shrub and trees (*P* = 0.04448) (Pearson’s chi-squared tests). No cattle behaviours were observed in dense shrub

Linking the behaviours observed with habitat, we found a significant difference in behaviour distribution between cattle and horses in conifer forest (*P* = 0.03), mixed and broadleaved forest (*P* = 0.03), open—dry heath and dunes (*P* = 0.03) and scattered shrub and trees (*P* = 0.04) (Pearson’s Chi-squared test) (Fig. 6). Furthermore, we observed minimal browsing behaviour in horses and cattle. We found that the dominant behaviour for cattle in conifer forest was grazing and standing, while horses were only observed standing. We found that grazing behaviour in horses was predominately observed in open habitats (heath-dune, wet, grassland and wetland), and to a lesser extent in scattered shrub and mixed woodland.

## Discussion

To support the increasing implementation of trophic rewilding through the introduction of large herbivores, it is essential to understand the potential for ecological effects across rewilding sites and ecosystems. Here, we provide insight into habitat use of semi-feral horses and cattle across multiple sites, characterising their space use within extensively grazed systems managed under trophic rewilding principles.

Animal movement occurs in structured patterns shaped by social organisation, perceived risk, and landscape connectivity; this means that the assumptions of ideal free distribution (Fretwell & Lucas, 1969) are unlikely to be met (Matthiopoulos et al., 2023). Within fenced rewilding sites, movement is further constrained, preventing dispersal beyond site limits. Ongoing population management, limited natural social structure, and fencing therefore likely result in space-use patterns that deviate from those expected under wild conditions.

Importantly, population densities at our 10 rewilding sites were mostly constant over time, except for the breeding horse populations at Langeland and Bøtø South, where densities fluctuated due to reproduction and population management. This suggests that observed habitat use was not primarily driven by changes in population density. The dominant drivers of observed space use patterns in our rewilding sites are likely to be resource-related, particularly food availability, modulated by species-specific foraging strategies and digestive constrains associated with hindgut fermentation (horses) versus rumination (cattle), and potentially further influenced by accessibility (e.g. dense vegetation), microclimatic conditions (e.g. shelter, shade) and inferred perceived predator risk.

To further interpret the observed habitat use patterns, we included behavioural observations. Behavioural allocation differed markedly between horses and cattle, consistent with what we can expect from their contrasting digestive physiology. Horses, as hindgut fermenters, spent more time actively grazing to meet nutritional requirements, whereas cattle spent less time grazing and more time resting and ruminating, reflecting constraints associated with rumen digestion, as ruminants are constrained by rumen capacity and fermentation time and therefore require a prolonged time to digest (Forbes & Kerley, 2022). These behavioural observations are consistent with previous studies of semi-feral and free-ranging cattle and horses in Europe, particularly in relation to behavioural time budgets and habitat use (Lamoot et al., 2005; Menard et al., 2002; Popp & Scheibe, 2014; Pratt et al., 1986; Putman et al., 1987). These contrasting behavioural strategies are expected to generate spatially heterogeneous patterns of habitat use across ecosystems.

Overall, across our sites, patterns of use varied across habitat types, with open habitats generally receiving higher use than structurally complex forest habitats. Wetlands were generally used less by cattle and horses than expected given availability, consistent with previous studies in European systems with semi-feral cattle (Pratt et al., 1986; Tofastrud et al., 2019) and horses (Cornelissen & Vulink, 2001; Cornelissen et al., 2014). However, both species increased wetland use during summer, likely reflecting increased forage availability and requirement for water resources (and potentially thermoregulation), supporting the interpretation that habitat use is dynamically linked to seasonal variation in resource distribution (Ebrahimi et al., 2010; Menard et al., 2002; Pratt et al., 1986).

In contrast to open habitats the forested habitats, particularly dense conifer stands and dense shrub, were least used by both cattle and horses. This pattern of low forest use is consistent with previous studies of wild and semi-wild horse populations in Europe (Chodkiewicz et al., 2023; Popp & Scheibe, 2014; Pratt et al., 1986) and North America (Leverkus et al., 2018; Schoenecker et al., 2023) and similarly for cattle (Popp & Scheibe, 2014; Spedener et al., 2024). Several factors may explain these patterns, including physical barriers to movement by dense vegetation (Leverkus et al., 2018) and reduced herbaceous vegetation in the understory caused by dense canopy cover.

However, previous studies have reported seasonal variation with increased woodland use during winter, when forage availability in open habitats declines and horse and cattle may shift towards browsing woody vegetation (Garrido et al., 2019; Hagstrup et al., 2021; Thomassen et al., 2023). We however did not observe a general increase in woodland use during winter and did not observe much browsing behaviour. Within conifer forests, cattle were primarily observed grazing, whereas horses were more frequently observed resting consistent with previous studies (Popp & Scheibe, 2014). Conversely, we observed cattle and horses using forests more during summer, suggesting that forests function as thermal refugia, with dense stands providing cooler microclimatic conditions compared to open habitats (Kemppinen et al., 2024) with a potential further role as shelter. Importantly, the relative low use of forest habitats across the 10 sites likely reflects the management legacy of the study sites, which are characterised by dense forestry plantations dominated by coniferous species with low palatability, sparce understory and consequently limited forage availability.

Linking behavioural observations to habitat use, we found that grazing was the dominant behaviour for horse and cattle in grasslands, open wet and heath-dune habitats, consistent with previous European studies on horse and cattle behaviour (Pratt et al., 1986; Putman et al., 1987). However, differences in digestive physiology imply contrasting spatial ecological effects. Cattle, due to longer resting and rumination periods, are likely to generate more spatially concentrated impacts, including trampling and bare soil formation in resting areas. In contrast, horses move continuously while grazing (Forbes & Kerley, 2022) and distributing grazing pressure more evenly across landscapes. Due to these differences, when having both species within a site, we can expect horse and cattle to generate complementary and spatially heterogeneous ecosystem effects across habitats and sites.

These patterns demonstrate that habitat composition and configuration can strongly shape herbivore habitat use, with immediate implications for predicting where grazing pressure, trampling, and nutrient redistribution will be concentrated in rewilding landscapes. Importantly, habitat use patterns may also translate into longer-term ecological effects. In a previous study on Danish natural areas, year-round grazing was associated with an increase in plant species richness and conservation value in dry grassland habitats, whereas a reduction in plant species richness were found in wetland habitats (Bergin et al., 2025). Together, these findings suggest that spatial patterns of herbivore habitat use can help explain variation in ecosystem responses across habitat types, linking movement ecology directly to biodiversity outcomes.

Furthermore, our results showed that management interventions can strongly influence habitat use patterns. Specifically, supplementary winter feeding appeared capable of substantially altering habitat use. In particular, the shifts observed in cattle indicate that localised feed provisioning may in effect concentrate animal activity in specific habitat types. This is important because such management may override otherwise emerging habitat-driven patterns of space use and thereby influence where grazing, trampling, and nutrient inputs are concentrated. This mirrors findings by Kristensen et al. (2026), who showed that artificial shelter similarly concentrated space use in a Danish rewilding area, underscoring the broader point that any localised infrastructure can override habitat-driven patterns of movement. While targeted attraction of herbivores may be useful in certain management contexts (e.g. reducing undesirable vegetation through trampling or grazing), such interventions contrast with the goals of trophic rewilding, which aim to promote self-organising ecological processes. By directing animal activity, these approaches may reduce the natural dynamics that rewilding seeks to restore (Bakker & Svenning, 2018). Supplementary feeding should therefore be considered explicitly when interpreting habitat-use patterns and when evaluating ecological outcomes in rewilding sites.

Thus, our results show that the expected ecological effects of animals in trophic rewilding projects depend strongly on site planning and management decisions, including habitat composition within fenced sites. In sites dominated by conifer plantations, we may find effects of animals to be pronounced in open habitats and minimal in forests, resulting in low site rewilding progress and overall reduced success (Mata et al., 2021). However, offering shade and dry ground in dense forests, is essential in the context of animal welfare requirements for domestic and kept semi-feral ungulates (Council of the European Union, 1998; Minister of Food, Agriculture and Fisheries, 2020) and is important to enable successful rewilding projects. In existing projects, site managers may consider to open up densely forested areas within sites, in order to enable increased ecological effects of animals throughout the site. For example, horses are found to significantly browse more on woody vegetation in open forests exposed to sunlight (Klich & Grudzień, 2013) and forest lawns and edges are often used by cattle (Tofastrud et al., 2018) and horses (Pratt et al., 1986).

Increasing the diversity of large herbivores may further enhance ecological effects. Forest-associated deer species, including browsers such as roe deer (*Capreolus capreolus*) and intermediate feeders such as red deer (*Cervus elaphus*) may increase browsing pressure on woody vegetation (Hofmann, 1989), while moose (*Alces alces*) and European bison (*Bison bonasus*) may significantly increase structural impacts in forests and wetlands (Kowalczyk et al., 2021). European bison in particular can strongly modify forest structure through bark stripping in conifer-dominated systems (Cromsigt et al., 2018; Hartvig et al., 2021; Nieszała et al., 2022).

Importantly, our results highlight that the initial habitat composition of sites is a critical factor to consider when planning the introduction of large herbivores in rewilding projects. A mix of habitats may be relevant for animals at different times of the year due to seasonal conditions (King & Gurnell, 2010). However, a large proportion of conifer plantation may lock the development and delay the potential for ecological effects and increased natural dynamics throughout a site. Therefore, relying on passive rewilding only (Corlett, 2016; Pettorelli et al., 2018; Svenning et al., 2016) for restoring ecosystems in past conifer plantations may considerably slow down and hinder the process of promoting ecological processes and achieving successful rewilding. Hence, site legacy should be a primary consideration during site planning, as it may affect the site potential and influence the magnitude of the effectiveness of restoration efforts (Cuddington, 2011; Slodowicz et al., 2023).

Our results have important implications for broader-scale implementation of trophic rewilding, because they show that site context strongly shapes the scope for herbivore-driven ecological processes. Across the studied systems, horses and cattle showed higher relative use of open habitats and comparatively limited use of dense shrub and conifer-dominated stands. This suggests that the ecological influence of these herbivores is likely to be distributed unevenly across habitats, with strongest effects in open parts of the sites. Consequently, rewilding trajectories depend not only on the introduced herbivores but also on initial habitat composition and landscape legacy. In sites dominated by dense plantation forest, horses and cattle may have limited capacity to generate broad landscape-scale restructuring in the short-term, implying that such sites may offer more constrained conditions for the emergence of self-organising trophic dynamics.

While forest habitats remain important parts of the ecosystem and for animal shelter and thermal refuge, these findings indicate that passive rewilding alone may be insufficient in some contexts. In such cases, limited initial structural interventions, such as partial opening of dense plantation stands, may in some contexts help create conditions under which herbivore-driven dynamics can develop more broadly across the site. Furthermore, incorporating knowledge of herbivore space use into site selection and long-term evaluation should improve understanding of where and how trophic rewilding is likely to generate ecological change and initiate the herbivore-driven spatial variability in herbivory, vegetation structure, and associated ecological processes that are characteristic of self-regulating, functionally complex ecosystems (Svenning et al., 2024). Together with careful site selection, for example using rewilding site assessment frameworks to identify most suitable rewilding using quantitative scores (Bergin et al., 2024) and knowledge of herbivore habitat use, this can support the development of more effective and successful rewilding projects.

## Conclusion

Large herbivores are central to trophic rewilding as through their uneven space use and selective feeding patterns (Kristensen et al., 2026; Ebrahimi et al., 2010) they promote more spatially and temporally heterogenous and functionally diverse ecological effects (Bakker et al., 2016; Oene et al., 1999; Trepel et al., 2024).

Across ten Danish rewilding sites, we found that semi-feral horses and cattle showed clear and consistent non-random habitat use, with both species exhibiting higher relative use of open habitats, especially grasslands, open wet habitats, and heath–dune vegetation, and lower relative use of dense shrub and conifer-dominated habitats. Habitat use also varied seasonally, with increased use of wet habitats in summer, while supplementary winter feeding may be altering the expected habitat use. These patterns indicate that habitat use is strongly linked to resource availability and accessibility within sites. The observed species-specific differences in habitat use and behaviour allocation highlight their functional complementarity. Assemblages including both species are therefore likely to generate more spatially and temporal heterogenous and functional diverse ecological effects, enhancing rewilding outcomes by supporting greater functional complexity.

Our results show that habitat composition, habitat structure, and management strongly shape the short-term and local distribution of herbivore activity within rewilding sites. Accordingly, the initial spatial pattern and rate of herbivore-driven ecological change can vary markedly among sites. In particular, sites dominated by dense conifer stands may offer more limited scope for horses and cattle to exert broad spatial effects than structurally more open and heterogeneous landscapes. Realising stronger ecological effects in such habitats may require structural interventions, such as canopy opening and the inclusion of additional browsing herbivore species, such as European bison.

Incorporating knowledge of herbivore space use into site selection, interpretation, and long-term evaluation should improve understanding of where and how trophic rewilding is likely to generate ecological change and promote more heterogeneous, self-organising ecosystems.

## Supplementary Information

Below is the link to the electronic supplementary material.

Supplementary file1 (DOCX 624 KB)

## Data Availability

The data that is generated and supports the findings of this study are available in the Supplementary Information of this article. The primary data and R scripts used in this manuscript are available on request.
