# Supplementary material for: Open habitats and species differences shape space use in semi-feral cattle and horses across Danish rewilding sites
Source: Environ Monit Assess. 2026 May 23;198(6):639. doi: 10.1007/s10661-026-15487-8 (PMC13197323; doi:10.1007/s10661-026-15487-8)
Supplement: Supplementary file 1 — Supplementary file1 (DOCX 624 KB) [file 10661_2026_15487_MOESM1_ESM.docx]

## Supplementing Information

Table S1 Habitats across all sites were mapped into eight categorical discrete habitat types. We ranked the sites according to an simplistic expected average availability of forage from low to high.

| **Productivity and available forage** | **Habitat type** | **Description** |
| --- | --- | --- |
| High  Low | Wetland | Bog habitats and open water. Water logged habitats. |
|  | Open wet | Meadows, grassland in more wet and nutrient rich soils. Include previous arable fields. |
|  | Open dry grassland | Grassland habitats on poorer soils, include previous arable and forestry sites. |
|  | Heath and dune | Heath an coastal dune habitats |
|  | Scattered shrub and trees | Mosaic of shrub and open habitats with occasional scattered trees. |
|  | Mixed broadleaved and conifer forest | Forest with broadleaved species, can have individual conifers within the forest stand. Most often plantations. |
|  | Conifer forest | Conifer plantations, often very dense pine (*Pinus* spp.), spruce (*Picea* spp.) and fir (*Abies* spp.) |
|  | Dense shrub | Low dense shrub (<3 m height), closed-canopy. Thickets of shrubs and young trees often pine (*Pinus* spp.) and thorn (*Prunus* spp). |

Table S2 Overview of study areas (Site) and number of GPS tracking locations collects (n) per individual (Animal ID) of horse and cattle (Group)

| **Site** | **Animal ID** | **Group** | **n** |
| --- | --- | --- | --- |
| Aal | AAC01 | Cattle | 4323 |
| Aal | AAC02 | Cattle | 5728 |
| Boto_north | BNH01 | Horse | 9806 |
| Boto_south | BSH01 | Horse | 9021 |
| Husbjerg | FUC01 | Cattle | 4168 |
| Husby | HBC01 | Cattle | 2080 |
| Husby | HBC02 | Cattle | 1049 |
| Husby | HBH01 | Horse | 2875 |
| Husby | HBH02 | Horse | 6014 |
| Klitmoller | KMH01 | Horse | 1886 |
| Klitmoller | KMH02 | Horse | 5644 |
| Laesoe | LSC01 | Cattle | 730 |
| Laesoe | LSC02 | Cattle | 1565 |
| Langeland | LLC01 | Cattle | 3386 |
| Langeland | LLH01 | Horse | 1396 |
| Langeland | LLH02 | Horse | 1115 |
| Langeland | LLH03 | Horse | 1529 |
| Langeland | LLH04 | Horse | 443 |
| Langeland | LLH05 | Horse | 6961 |
| Langeland | LLH06 | Horse | 3245 |
| Langeland | LLH07 | Horse | 1169 |
| Langeland | LLH08 | Horse | 5423 |
| Langeland | LLH09 | Horse | 6228 |
| Ulvshale_north | UNH01 | Horse | 5197 |
| Ulvshale_south | USC01 | Cattle | 7125 |
| Ulvshale_south | USC03 | Cattle | 788 |
| Ulvshale_south | USH01 | Horse | 7569 |

Table S3 Seasonal GPS-data points (n) per site for horses and cattle (Group)

| **Site** | **Group** | **Season** | **n** |
| --- | --- | --- | --- |
| Aal | Cattle | Autumn | 1910 |
| Aal | Cattle | Spring | 3938 |
| Aal | Cattle | Summer | 3151 |
| Aal | Cattle | Winter | 1052 |
| Boto_north | Horse | Autumn | 797 |
| Boto_north | Horse | Spring | 4221 |
| Boto_north | Horse | Summer | 3126 |
| Boto_north | Horse | Winter | 1662 |
| Boto_south | Horse | Autumn | 2074 |
| Boto_south | Horse | Spring | 2140 |
| Boto_south | Horse | Summer | 2136 |
| Boto_south | Horse | Winter | 2671 |
| Husbjerg | Cattle | Autumn | 751 |
| Husbjerg | Cattle | Spring | 1517 |
| Husbjerg | Cattle | Summer | 589 |
| Husbjerg | Cattle | Winter | 1311 |
| Husby | Cattle | Autumn | 413 |
| Husby | Cattle | Spring | 737 |
| Husby | Cattle | Summer | 734 |
| Husby | Cattle | Winter | 1245 |
| Husby | Horse | Autumn | 2278 |
| Husby | Horse | Spring | 1524 |
| Husby | Horse | Summer | 3454 |
| Husby | Horse | Winter | 1633 |
| Klitmoller | Horse | Autumn | 1650 |
| Klitmoller | Horse | Spring | 1959 |
| Klitmoller | Horse | Summer | 3014 |
| Klitmoller | Horse | Winter | 907 |
| Laesoe | Cattle | Autumn | 364 |
| Laesoe | Cattle | Spring | 778 |
| Laesoe | Cattle | Summer | 993 |
| Laesoe | Cattle | Winter | 160 |
| Langeland | Cattle | Autumn | 608 |
| Langeland | Cattle | Spring | 835 |
| Langeland | Cattle | Summer | 1785 |
| Langeland | Cattle | Winter | 158 |
| Langeland | Horse | Autumn | 4526 |
| Langeland | Horse | Spring | 9229 |
| Langeland | Horse | Summer | 6152 |
| Langeland | Horse | Winter | 7738 |
| Risbaek | Horse | Autumn | 1058 |
| Risbaek | Horse | Spring | 1632 |
| Risbaek | Horse | Summer | 1491 |
| Risbaek | Horse | Winter | 1460 |
| Ulvshale_north | Horse | Autumn | 1407 |
| Ulvshale_north | Horse | Spring | 1394 |
| Ulvshale_north | Horse | Summer | 1080 |
| Ulvshale_north | Horse | Winter | 1316 |
| Ulvshale_south | Cattle | Autumn | 2836 |
| Ulvshale_south | Cattle | Spring | 1077 |
| Ulvshale_south | Cattle | Summer | 2909 |
| Ulvshale_south | Cattle | Winter | 1091 |
| Ulvshale_south | Horse | Autumn | 2129 |
| Ulvshale_south | Horse | Spring | 1865 |
| Ulvshale_south | Horse | Summer | 1877 |
| Ulvshale_south | Horse | Winter | 1698 |

Table S4 Categorising behaviour observed in semi-feral horse and cattle across study sites in Denmark.

| **Behaviour** | **Grazing** | **Browsing** | **Drinking** | **Standing** | **Resting** | **Walking** | **Lactating** | **Rubbing** | **Wallowing** | **Other** |
| --- | --- | --- | --- | --- | --- | --- | --- | --- | --- | --- |
| **Description** | Grazing ,moving with head down | Browsing on twigs, leafs and buds | Drinking from natural water sites | Standing, resting. doing nothing else | Lying, include cattle ruminating | Moving, including running | Foals drinking from mother | Rubbing body against object or biting/scratching. | Laying or rolling in mud, water or soft ground |  |


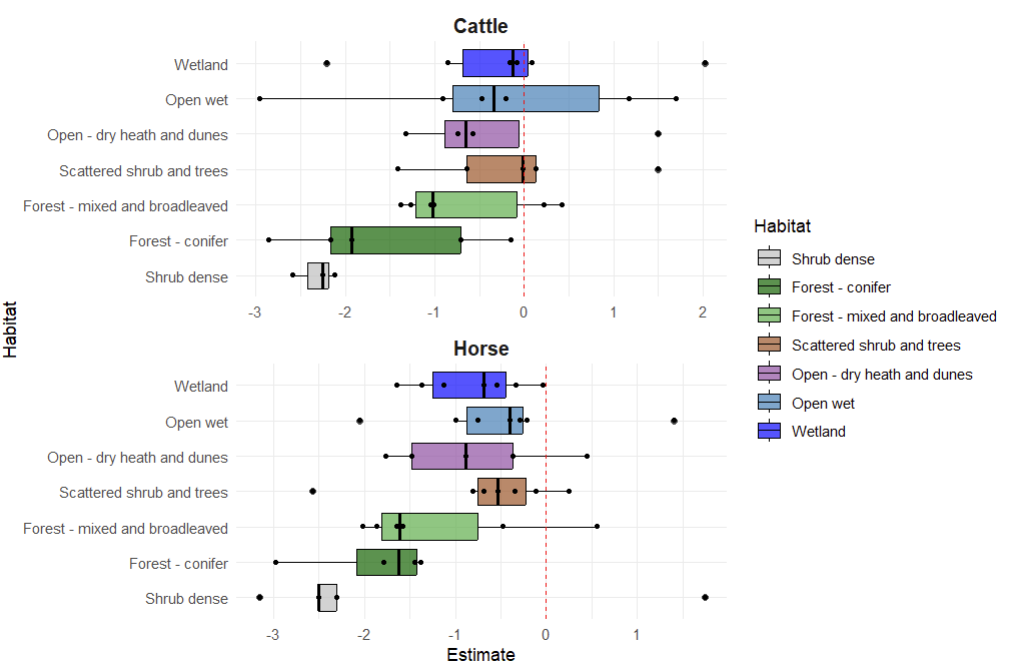


Figure S5 Habitat selection coefficients (Estimate) displayed in order of density of vegetation and expected forage availability. The study was based on GPS location data from cattle (N=6) and horses (N=7) and analysed using resource selection functions – the boxplots summarise individual selection coefficients. Data from ten study sites across Denmark, where cattle and horses co-habited at three sites: Husby, Langeland and Ulvshale South. Data spanned for up to two years, and this analysis did not take season in to account. A selection coefficient of O represents an equal use-availability ratio to the reference category habitat, open dry grassland. Conifer forest (dark green) was avoided by cattle and horses, as was dense shrub (grey), and to a lesser extent mixed and broadleaved forest (light green). Cattle selected for wetland (blue), open wet (light blue) and scattered shrub (brown) to a greater extent than horses, and overall horses select for grassland above all other habitats. All selection coefficients are significantly different to grassland apart from four animal x habitat combinations.

Table S6 The relative number of observations in dry grassland compared to other habitat (range where more animals tagged per site per).

| Site | Bøtø North | Bøtø South | Husbjerg | Husby | Husby | Klitmøller | Langeland | Langeland | Læsø | Ulvshale North | Ulvshale South | Ulvshale South | All |
| --- | --- | --- | --- | --- | --- | --- | --- | --- | --- | --- | --- | --- | --- |
| Group (animal) | **Horse** | **Horse** | **Cattle** | **Cattle** | **Horse** | **Horse** | **Cattle** | **Horse** | **Cattle** | **Horse** | **Cattle** | **Horse** | **Cattle** |
| Forest - conifer |  |  | 32.32 | 1.49-1.53 | 0.7-1.3 | 0.11-0.20 | 284.22 | 66-408 | 44220 | 18.42 | 5.8 |  | 0.72-1.40 |
| Forest - mixed and broadleaved | 0.89 | 0.47 | 1.28 | 2.60-3.34 | 4.03-5.85 |  | 12.23 | 9-21 | 0.00 | 2.80 |  | 3.41 | 0.93-1.50 |
| Open - dry heath and dunes |  | 26.04 | 5.82 | 0.5-0.75 | 0.47-.049 | 0.01-0.02 |  |  | 0.00 | 0.64 | 1.41 | 1.60 | 10.86- 17.51 |
| Open wet | 0.02 | 0.78 |  | 0.25-0.27 | 0.12-0.19 | 0.42-0.58 | 9.50 | 4.49-9.50 | 0.02 | 0.61 | 0.12 | 0.11 | 3.36-13.95 |
| Scattered shrub and trees | 0.11 | 0.35 |  | 0.62.0.70 | 0.80-1.11 | 0.07-0.11 | 13.46 | 13.20-22.63 | 0.02 | 2.48 | 2.27 | 2.65 | 5.06-12.22 |
| Shrub dense | 2.07 |  |  |  |  | 0.17-0.34 | 319.75 | 208-859 |  | 76.53 | 17.72 | 13.08 | 75.28-218.9 |
| Wetland | 0.14 | 24.96 | 35.06 | 4.59-10.18 | 3.42-5.57 | 0.41-1.63 | 17.88 | 17-30(338) | 0.05 | 6.29 | 1.79 | 1.98 | 21.08-56.12 |

Table S7 Resource selection coefficient (Estimate) and derived Exponential selection coefficient/ relative selection strength (expCoef) and area-adjusted selection coefficient (exp_Coef_adj) from 27 animals (Horses and cattle) across 10 sites in Denmark. The selection coefficients are the use:availability ratio of each habitat in relation to the use:availability ratio of the reference habitat grassland. of observations in grassland to other habitat (range per site and group). The relative use of grassland compared to various habitat (Rel use grassland).

| **Animal ID** | **Estimate** | **Std. Error** | **Z value** | **P-Value** | **Exp Coef** | **N**  **avail** | **N avail**  **total** | **Prop**  **Avail** | **Exp**  **Coef**  **adj** | **Habitat** | **Rel use** | **Site** | **Group** |
| --- | --- | --- | --- | --- | --- | --- | --- | --- | --- | --- | --- | --- | --- |
|  |  |  |  |  |  |  |  |  |  |  | **grassland** |  |  |
| BNH01 | 0.555 | 0.112 | 4.96 | 0.000 | 1.7414 | 1640 | 195574 | 0.008 | 1.154 | Forest - mixed and broadleaved | 0.866 | Boto_north | Horse |
| BNH01 | -11.327 | 0.082 | -138.26 | 0.000 | 0.0000 | 2474 | 195574 | 0.013 | NA | Open - dry grassland | 1.000 | Boto_north | Horse |
| BNH01 | -0.214 | 0.083 | -2.59 | 0.010 | 0.8071 | 141928 | 195574 | 0.726 | 46.302 | Open wet | 0.022 | Boto_north | Horse |
| BNH01 | 0.249 | 0.086 | 2.89 | 0.004 | 1.2833 | 17958 | 195574 | 0.092 | 9.315 | Scattered shrub and trees | 0.107 | Boto_north | Horse |
| BNH01 | 1.739 | 0.144 | 12.12 | 0.000 | 5.6928 | 210 | 195574 | 0.001 | 0.483 | Shrub dense | 2.069 | Boto_north | Horse |
| BNH01 | -0.546 | 0.087 | -6.25 | 0.000 | 0.5792 | 31364 | 195574 | 0.160 | 7.342 | Wetland | 0.136 | Boto_north | Horse |
| BSH01 | -1.579 | 0.035 | -45.07 | 0.000 | 0.2062 | 105796 | 176648 | 0.599 | 2.124 | Forest - mixed and broadleaved | 0.471 | Boto_south | Horse |
| BSH01 | -10.666 | 0.029 | -369.15 | 0.000 | 0.0000 | 10267 | 176648 | 0.058 | NA | Open - dry grassland | 1.000 | Boto_south | Horse |
| BSH01 | 0.444 | 0.150 | 2.95 | 0.003 | 1.5582 | 253 | 176648 | 0.001 | 0.038 | Open - dry heath and dunes | 26.043 | Boto_south | Horse |
| BSH01 | -0.295 | 0.039 | -7.66 | 0.000 | 0.7446 | 17749 | 176648 | 0.100 | 1.287 | Open wet | 0.777 | Boto_south | Horse |
| BSH01 | -0.345 | 0.034 | -10.28 | 0.000 | 0.7084 | 41761 | 176648 | 0.236 | 2.881 | Scattered shrub and trees | 0.347 | Boto_south | Horse |
| BSH01 | -0.692 | 0.147 | -4.70 | 0.000 | 0.5004 | 822 | 176648 | 0.005 | 0.040 | Wetland | 24.958 | Boto_south | Horse |
| FUC01 | -0.145 | 0.127 | -1.14 | 0.254 | 0.8651 | 1660 | 83220 | 0.020 | 0.031 | Forest - conifer | 32.328 | Husbjerg | Cattle |
| FUC01 | 0.419 | 0.033 | 12.61 | 0.000 | 1.5201 | 23825 | 83220 | 0.286 | 0.780 | Forest - mixed and broadleaved | 1.282 | Husbjerg | Cattle |
| FUC01 | -11.628 | 0.022 | -528.91 | 0.000 | 0.0000 | 46426 | 83220 | 0.558 | NA | Open - dry grassland | 1.000 | Husbjerg | Cattle |
| FUC01 | -0.202 | 0.057 | -3.51 | 0.000 | 0.8172 | 9748 | 83220 | 0.117 | 0.172 | Open wet | 5.828 | Husbjerg | Cattle |
| FUC01 | -0.165 | 0.132 | -1.25 | 0.212 | 0.8481 | 1561 | 83220 | 0.019 | 0.029 | Wetland | 35.068 | Husbjerg | Cattle |
| HBC01 | -1.930 | 0.104 | -18.56 | 0.000 | 0.1452 | 15497 | 41440 | 0.374 | 0.654 | Forest - conifer | 1.529 | Husby | Cattle |
| HBC01 | -1.048 | 0.136 | -7.70 | 0.000 | 0.3505 | 2937 | 41440 | 0.071 | 0.299 | Forest - mixed and broadleaved | 3.343 | Husby | Cattle |
| HBC01 | -11.205 | 0.065 | -171.41 | 0.000 | 0.0000 | 3441 | 41440 | 0.083 | NA | Open - dry grassland | 1.000 | Husby | Cattle |
| HBC01 | -0.738 | 0.086 | -8.53 | 0.000 | 0.4781 | 9597 | 41440 | 0.232 | 1.333 | Open - dry heath and dunes | 0.750 | Husby | Cattle |
| HBC01 | 1.178 | 0.073 | 16.08 | 0.000 | 3.2481 | 4147 | 41440 | 0.100 | 3.915 | Open wet | 0.255 | Husby | Cattle |
| HBC01 | -0.013 | 0.085 | -0.15 | 0.880 | 0.9872 | 5005 | 41440 | 0.121 | 1.436 | Scattered shrub and trees | 0.696 | Husby | Cattle |
| HBC01 | -0.084 | 0.155 | -0.55 | 0.585 | 0.9191 | 816 | 41440 | 0.020 | 0.218 | Wetland | 4.588 | Husby | Cattle |
| HBC02 | -1.920 | 0.149 | -12.87 | 0.000 | 0.1466 | 7840 | 20760 | 0.378 | 0.670 | Forest - conifer | 1.493 | Husby | Cattle |
| HBC02 | -0.788 | 0.179 | -4.39 | 0.000 | 0.4547 | 1449 | 20760 | 0.070 | 0.384 | Forest - mixed and broadleaved | 2.605 | Husby | Cattle |
| HBC02 | -11.246 | 0.094 | -119.02 | 0.000 | 0.0000 | 1716 | 20760 | 0.083 | NA | Open - dry grassland | 1.000 | Husby | Cattle |
| HBC02 | -0.415 | 0.117 | -3.54 | 0.000 | 0.6603 | 4803 | 20760 | 0.231 | 1.848 | Open - dry heath and dunes | 0.541 | Husby | Cattle |
| HBC02 | 1.104 | 0.107 | 10.35 | 0.000 | 3.0170 | 2072 | 20760 | 0.100 | 3.643 | Open wet | 0.275 | Husby | Cattle |
| HBC02 | 0.131 | 0.120 | 1.09 | 0.274 | 1.1405 | 2445 | 20760 | 0.118 | 1.625 | Scattered shrub and trees | 0.615 | Husby | Cattle |
| HBC02 | -0.948 | 0.316 | -3.00 | 0.003 | 0.3874 | 435 | 20760 | 0.021 | 0.098 | Wetland | 10.182 | Husby | Cattle |
| HBH01 | -1.201 | 0.092 | -13.02 | 0.000 | 0.3009 | 21373 | 57081 | 0.374 | 1.391 | Forest - conifer | 0.719 | Husby | Horse |
| HBH01 | -1.248 | 0.158 | -7.91 | 0.000 | 0.2869 | 3988 | 57081 | 0.070 | 0.248 | Forest - mixed and broadleaved | 4.040 | Husby | Horse |
| HBH01 | -11.648 | 0.070 | -165.55 | 0.000 | 0.0000 | 4623 | 57081 | 0.081 | NA | Open - dry grassland | 1.000 | Husby | Horse |
| HBH01 | -0.312 | 0.085 | -3.65 | 0.000 | 0.7319 | 13321 | 57081 | 0.233 | 2.109 | Open - dry heath and dunes | 0.474 | Husby | Horse |
| HBH01 | 1.840 | 0.075 | 24.64 | 0.000 | 6.2994 | 5762 | 57081 | 0.101 | 7.851 | Open wet | 0.127 | Husby | Horse |
| HBH01 | -0.183 | 0.095 | -1.93 | 0.053 | 0.8330 | 6869 | 57081 | 0.120 | 1.238 | Scattered shrub and trees | 0.808 | Husby | Horse |
| HBH01 | 0.165 | 0.148 | 1.11 | 0.265 | 1.1793 | 1145 | 57081 | 0.020 | 0.292 | Wetland | 3.424 | Husby | Horse |
| HBH02 | -1.792 | 0.062 | -28.72 | 0.000 | 0.1667 | 44686 | 119320 | 0.375 | 0.783 | Forest - conifer | 1.277 | Husby | Horse |
| HBH02 | -1.645 | 0.108 | -15.20 | 0.000 | 0.1929 | 8427 | 119320 | 0.071 | 0.171 | Forest - mixed and broadleaved | 5.850 | Husby | Horse |
| HBH02 | -11.306 | 0.041 | -273.45 | 0.000 | 0.0000 | 9512 | 119320 | 0.080 | NA | Open - dry grassland | 1.000 | Husby | Horse |
| HBH02 | -0.364 | 0.051 | -7.22 | 0.000 | 0.6946 | 27834 | 119320 | 0.233 | 2.032 | Open - dry heath and dunes | 0.492 | Husby | Horse |
| HBH02 | 1.407 | 0.045 | 31.13 | 0.000 | 4.0833 | 11962 | 119320 | 0.100 | 5.135 | Open wet | 0.195 | Husby | Horse |
| HBH02 | -0.531 | 0.060 | -8.83 | 0.000 | 0.5880 | 14517 | 119320 | 0.122 | 0.897 | Scattered shrub and trees | 1.114 | Husby | Horse |
| HBH02 | -0.333 | 0.106 | -3.14 | 0.002 | 0.7167 | 2382 | 119320 | 0.020 | 0.179 | Wetland | 5.571 | Husby | Horse |
| KMH01 | -2.170 | 0.259 | -8.39 | 0.000 | 0.1142 | 985 | 37720 | 0.026 | 4.889 | Forest - conifer | 0.205 | Klitmoller | Horse |
| KMH01 | -8.762 | 0.236 | -37.17 | 0.000 | 0.0002 | 23 | 37720 | 0.001 | NA | Open - dry grassland | 1.000 | Klitmoller | Horse |
| KMH01 | -3.342 | 0.350 | -9.56 | 0.000 | 0.0354 | 26882 | 37720 | 0.713 | 41.331 | Open - dry heath and dunes | 0.024 | Klitmoller | Horse |
| KMH01 | -2.614 | 0.237 | -11.02 | 0.000 | 0.0732 | 542 | 37720 | 0.014 | 1.726 | Open wet | 0.579 | Klitmoller | Horse |
| KMH01 | -3.259 | 0.249 | -13.11 | 0.000 | 0.0384 | 5321 | 37720 | 0.141 | 8.889 | Scattered shrub and trees | 0.113 | Klitmoller | Horse |
| KMH01 | -4.007 | 0.273 | -14.69 | 0.000 | 0.0182 | 3722 | 37720 | 0.099 | 2.944 | Shrub dense | 0.340 | Klitmoller | Horse |
| KMH01 | -2.858 | 0.383 | -7.47 | 0.000 | 0.0574 | 245 | 37720 | 0.006 | 0.611 | Wetland | 1.636 | Klitmoller | Horse |
| KMH02 | -1.378 | 0.183 | -7.53 | 0.000 | 0.2522 | 2934 | 112880 | 0.026 | 9.485 | Forest - conifer | 0.105 | Klitmoller | Horse |
| KMH02 | -9.377 | 0.174 | -53.87 | 0.000 | 0.0001 | 78 | 112880 | 0.001 | NA | Open - dry grassland | 1.000 | Klitmoller | Horse |
| KMH02 | -1.765 | 0.200 | -8.83 | 0.000 | 0.1712 | 81443 | 112880 | 0.722 | 178.739 | Open - dry heath and dunes | 0.006 | Klitmoller | Horse |
| KMH02 | -2.055 | 0.175 | -11.76 | 0.000 | 0.1281 | 1436 | 112880 | 0.013 | 2.358 | Open wet | 0.424 | Klitmoller | Horse |
| KMH02 | -2.565 | 0.180 | -14.27 | 0.000 | 0.0770 | 15388 | 112880 | 0.136 | 15.182 | Scattered shrub and trees | 0.066 | Klitmoller | Horse |
| KMH02 | -3.154 | 0.188 | -16.78 | 0.000 | 0.0427 | 11016 | 112880 | 0.098 | 6.030 | Shrub dense | 0.166 | Klitmoller | Horse |
| KMH02 | -1.129 | 0.207 | -5.46 | 0.000 | 0.3232 | 585 | 112880 | 0.005 | 2.424 | Wetland | 0.413 | Klitmoller | Horse |
| LLC01 | -2.855 | 0.333 | -8.56 | 0.000 | 0.0576 | 2214 | 67744 | 0.033 | 0.004 | Forest - conifer | 284.221 | Langeland | Cattle |
| LLC01 | -1.378 | 0.072 | -19.15 | 0.000 | 0.2521 | 11742 | 67744 | 0.173 | 0.082 | Forest - mixed and broadleaved | 12.239 | Langeland | Cattle |
| LLC01 | -11.168 | 0.020 | -564.83 | 0.000 | 0.0000 | 36227 | 67744 | 0.535 | NA | Open - dry grassland | 1.000 | Langeland | Cattle |
| LLC01 | -0.912 | 0.064 | -14.22 | 0.000 | 0.4019 | 9479 | 67744 | 0.140 | 0.105 | Open wet | 9.509 | Langeland | Cattle |
| LLC01 | 0.130 | 0.075 | 1.73 | 0.083 | 1.1392 | 2362 | 67744 | 0.035 | 0.074 | Scattered shrub and trees | 13.463 | Langeland | Cattle |
| LLC01 | -2.125 | 0.354 | -6.00 | 0.000 | 0.1194 | 949 | 67744 | 0.014 | 0.003 | Shrub dense | 319.750 | Langeland | Cattle |
| LLC01 | -0.857 | 0.086 | -9.97 | 0.000 | 0.4245 | 4771 | 67744 | 0.070 | 0.056 | Wetland | 17.888 | Langeland | Cattle |
| LLH01 | -2.144 | 0.379 | -5.65 | 0.000 | 0.1172 | 910 | 27926 | 0.033 | 0.007 | Forest - conifer | 141.429 | Langeland | Horse |
| LLH01 | -1.449 | 0.121 | -12.02 | 0.000 | 0.2349 | 4801 | 27926 | 0.172 | 0.075 | Forest - mixed and broadleaved | 13.378 | Langeland | Horse |
| LLH01 | -11.241 | 0.032 | -353.69 | 0.000 | 0.0000 | 15089 | 27926 | 0.540 | NA | Open - dry grassland | 1.000 | Langeland | Horse |
| LLH01 | -0.184 | 0.076 | -2.43 | 0.015 | 0.8319 | 3884 | 27926 | 0.139 | 0.214 | Open wet | 4.670 | Langeland | Horse |
| LLH01 | 0.129 | 0.125 | 1.03 | 0.304 | 1.1377 | 911 | 27926 | 0.033 | 0.069 | Scattered shrub and trees | 14.559 | Langeland | Horse |
| LLH01 | -1.722 | 0.501 | -3.44 | 0.001 | 0.1788 | 341 | 27926 | 0.012 | 0.004 | Shrub dense | 247.500 | Langeland | Horse |
| LLH01 | -1.158 | 0.159 | -7.27 | 0.000 | 0.3140 | 1990 | 27926 | 0.071 | 0.041 | Wetland | 24.146 | Langeland | Horse |
| LLH02 | -2.467 | 0.501 | -4.92 | 0.000 | 0.0848 | 721 | 22289 | 0.032 | 0.005 | Forest - conifer | 194.750 | Langeland | Horse |
| LLH02 | -1.164 | 0.118 | -9.86 | 0.000 | 0.3123 | 3867 | 22289 | 0.173 | 0.101 | Forest - mixed and broadleaved | 9.861 | Langeland | Horse |
| LLH02 | -11.244 | 0.036 | -313.83 | 0.000 | 0.0000 | 11907 | 22289 | 0.534 | NA | Open - dry grassland | 1.000 | Langeland | Horse |
| LLH02 | -0.198 | 0.086 | -2.31 | 0.021 | 0.8202 | 3075 | 22289 | 0.138 | 0.212 | Open wet | 4.721 | Langeland | Horse |
| LLH02 | 0.111 | 0.135 | 0.82 | 0.411 | 1.1175 | 807 | 22289 | 0.036 | 0.076 | Scattered shrub and trees | 13.203 | Langeland | Horse |
| LLH02 | -1.858 | 0.578 | -3.21 | 0.001 | 0.1560 | 294 | 22289 | 0.013 | 0.004 | Shrub dense | 259.667 | Langeland | Horse |
| LLH02 | -1.443 | 0.203 | -7.10 | 0.000 | 0.2362 | 1618 | 22289 | 0.073 | 0.032 | Wetland | 31.160 | Langeland | Horse |
| LLH03 | -1.574 | 0.279 | -5.64 | 0.000 | 0.2073 | 956 | 30578 | 0.031 | 0.012 | Forest - conifer | 83.385 | Langeland | Horse |
| LLH03 | -1.522 | 0.119 | -12.83 | 0.000 | 0.2183 | 5308 | 30578 | 0.174 | 0.070 | Forest - mixed and broadleaved | 14.263 | Langeland | Horse |
| LLH03 | -11.242 | 0.030 | -370.11 | 0.000 | 0.0000 | 16526 | 30578 | 0.540 | NA | Open - dry grassland | 1.000 | Langeland | Horse |
| LLH03 | -0.110 | 0.071 | -1.57 | 0.118 | 0.8955 | 4205 | 30578 | 0.138 | 0.228 | Open wet | 4.389 | Langeland | Horse |
| LLH03 | -0.124 | 0.132 | -0.94 | 0.345 | 0.8832 | 1053 | 30578 | 0.034 | 0.056 | Scattered shrub and trees | 17.770 | Langeland | Horse |
| LLH03 | -2.577 | 0.707 | -3.64 | 0.000 | 0.0760 | 401 | 30578 | 0.013 | 0.002 | Shrub dense | 542.000 | Langeland | Horse |
| LLH03 | -1.132 | 0.152 | -7.44 | 0.000 | 0.3222 | 2129 | 30578 | 0.070 | 0.042 | Wetland | 24.089 | Langeland | Horse |
| LLH04 | -1.352 | 0.451 | -3.00 | 0.003 | 0.2587 | 269 | 8863 | 0.030 | 0.015 | Forest - conifer | 67.800 | Langeland | Horse |
| LLH04 | -1.660 | 0.225 | -7.38 | 0.000 | 0.1902 | 1537 | 8863 | 0.173 | 0.062 | Forest - mixed and broadleaved | 16.143 | Langeland | Horse |
| LLH04 | -11.150 | 0.054 | -205.30 | 0.000 | 0.0000 | 4718 | 8863 | 0.532 | NA | Open - dry grassland | 1.000 | Langeland | Horse |
| LLH04 | -0.475 | 0.144 | -3.29 | 0.001 | 0.6220 | 1253 | 8863 | 0.141 | 0.165 | Open wet | 6.054 | Langeland | Horse |
| LLH04 | -0.172 | 0.236 | -0.73 | 0.466 | 0.8421 | 314 | 8863 | 0.035 | 0.056 | Scattered shrub and trees | 17.842 | Langeland | Horse |
| LLH04 | -1.650 | 0.709 | -2.33 | 0.020 | 0.1920 | 145 | 8863 | 0.016 | 0.006 | Shrub dense | 169.500 | Langeland | Horse |
| LLH04 | -3.808 | 1.001 | -3.81 | 0.000 | 0.0222 | 627 | 8863 | 0.071 | 0.003 | Wetland | 338.999 | Langeland | Horse |
| LLH05 | -2.973 | 0.242 | -12.26 | 0.000 | 0.0511 | 4521 | 139287 | 0.032 | 0.003 | Forest - conifer | 321.586 | Langeland | Horse |
| LLH05 | -1.863 | 0.062 | -30.15 | 0.000 | 0.1552 | 24103 | 139287 | 0.173 | 0.050 | Forest - mixed and broadleaved | 19.880 | Langeland | Horse |
| LLH05 | -11.127 | 0.014 | -822.75 | 0.000 | 0.0000 | 74365 | 139287 | 0.534 | NA | Open - dry grassland | 1.000 | Langeland | Horse |
| LLH05 | -0.748 | 0.040 | -18.51 | 0.000 | 0.4735 | 19849 | 139287 | 0.143 | 0.126 | Open wet | 7.912 | Langeland | Horse |
| LLH05 | -0.114 | 0.058 | -1.98 | 0.047 | 0.8919 | 4850 | 139287 | 0.035 | 0.058 | Scattered shrub and trees | 17.192 | Langeland | Horse |
| LLH05 | -2.507 | 0.302 | -8.31 | 0.000 | 0.0815 | 1835 | 139287 | 0.013 | 0.002 | Shrub dense | 497.000 | Langeland | Horse |
| LLH05 | -1.372 | 0.075 | -18.21 | 0.000 | 0.2535 | 9764 | 139287 | 0.070 | 0.033 | Wetland | 30.038 | Langeland | Horse |
| LLH06 | -1.700 | 0.201 | -8.46 | 0.000 | 0.1827 | 2068 | 64882 | 0.032 | 0.011 | Forest - conifer | 91.840 | Langeland | Horse |
| LLH06 | -1.372 | 0.075 | -18.17 | 0.000 | 0.2537 | 11318 | 64882 | 0.174 | 0.083 | Forest - mixed and broadleaved | 12.084 | Langeland | Horse |
| LLH06 | -11.233 | 0.021 | -538.23 | 0.000 | 0.0000 | 34700 | 64882 | 0.535 | NA | Open - dry grassland | 1.000 | Langeland | Horse |
| LLH06 | -0.176 | 0.049 | -3.60 | 0.000 | 0.8386 | 9209 | 64882 | 0.142 | 0.223 | Open wet | 4.493 | Langeland | Horse |
| LLH06 | -0.198 | 0.092 | -2.15 | 0.032 | 0.8205 | 2284 | 64882 | 0.035 | 0.054 | Scattered shrub and trees | 18.516 | Langeland | Horse |
| LLH06 | -1.640 | 0.302 | -5.43 | 0.000 | 0.1940 | 857 | 64882 | 0.013 | 0.005 | Shrub dense | 208.727 | Langeland | Horse |
| LLH06 | -1.230 | 0.110 | -11.20 | 0.000 | 0.2923 | 4446 | 64882 | 0.069 | 0.037 | Wetland | 26.698 | Langeland | Horse |
| LLH07 | -1.419 | 0.279 | -5.08 | 0.000 | 0.2419 | 778 | 23388 | 0.033 | 0.015 | Forest - conifer | 66.154 | Langeland | Horse |
| LLH07 | -1.584 | 0.135 | -11.77 | 0.000 | 0.2051 | 4164 | 23388 | 0.178 | 0.069 | Forest - mixed and broadleaved | 14.576 | Langeland | Horse |
| LLH07 | -11.190 | 0.034 | -328.14 | 0.000 | 0.0000 | 12448 | 23388 | 0.532 | NA | Open - dry grassland | 1.000 | Langeland | Horse |
| LLH07 | -0.342 | 0.086 | -3.96 | 0.000 | 0.7103 | 3240 | 23388 | 0.139 | 0.185 | Open wet | 5.409 | Langeland | Horse |
| LLH07 | -0.375 | 0.166 | -2.26 | 0.024 | 0.6875 | 800 | 23388 | 0.034 | 0.044 | Scattered shrub and trees | 22.632 | Langeland | Horse |
| LLH07 | -3.061 | 0.997 | -3.07 | 0.002 | 0.0468 | 309 | 23388 | 0.013 | 0.001 | Shrub dense | 859.981 | Langeland | Horse |
| LLH07 | -1.072 | 0.164 | -6.55 | 0.000 | 0.3423 | 1649 | 23388 | 0.071 | 0.045 | Wetland | 22.051 | Langeland | Horse |
| LLH08 | -3.216 | 0.315 | -10.21 | 0.000 | 0.0401 | 3537 | 108486 | 0.033 | 0.002 | Forest - conifer | 408.275 | Langeland | Horse |
| LLH08 | -1.923 | 0.073 | -26.17 | 0.000 | 0.1461 | 18832 | 108486 | 0.174 | 0.048 | Forest - mixed and broadleaved | 21.046 | Langeland | Horse |
| LLH08 | -11.169 | 0.016 | -713.70 | 0.000 | 0.0000 | 57915 | 108486 | 0.534 | NA | Open - dry grassland | 1.000 | Langeland | Horse |
| LLH08 | -0.388 | 0.040 | -9.64 | 0.000 | 0.6783 | 15202 | 108486 | 0.140 | 0.178 | Open wet | 5.616 | Langeland | Horse |
| LLH08 | 0.016 | 0.062 | 0.26 | 0.794 | 1.0163 | 3880 | 108486 | 0.036 | 0.068 | Scattered shrub and trees | 14.687 | Langeland | Horse |
| LLH08 | -2.324 | 0.317 | -7.34 | 0.000 | 0.0979 | 1449 | 108486 | 0.013 | 0.002 | Shrub dense | 408.300 | Langeland | Horse |
| LLH08 | -1.506 | 0.093 | -16.26 | 0.000 | 0.2219 | 7671 | 108486 | 0.071 | 0.029 | Wetland | 34.025 | Langeland | Horse |
| LLH09 | -2.001 | 0.170 | -11.79 | 0.000 | 0.1352 | 3930 | 124610 | 0.032 | 0.008 | Forest - conifer | 125.600 | Langeland | Horse |
| LLH09 | -1.408 | 0.056 | -25.25 | 0.000 | 0.2447 | 21537 | 124610 | 0.173 | 0.079 | Forest - mixed and broadleaved | 12.669 | Langeland | Horse |
| LLH09 | -11.238 | 0.015 | -745.07 | 0.000 | 0.0000 | 66758 | 124610 | 0.536 | NA | Open - dry grassland | 1.000 | Langeland | Horse |
| LLH09 | -0.162 | 0.035 | -4.59 | 0.000 | 0.8501 | 17453 | 124610 | 0.140 | 0.222 | Open wet | 4.499 | Langeland | Horse |
| LLH09 | -0.034 | 0.062 | -0.55 | 0.581 | 0.9664 | 4353 | 124610 | 0.035 | 0.063 | Scattered shrub and trees | 15.870 | Langeland | Horse |
| LLH09 | -2.122 | 0.278 | -7.64 | 0.000 | 0.1198 | 1648 | 124610 | 0.013 | 0.003 | Shrub dense | 338.154 | Langeland | Horse |
| LLH09 | -1.173 | 0.076 | -15.51 | 0.000 | 0.3095 | 8931 | 124610 | 0.072 | 0.041 | Wetland | 24.154 | Langeland | Horse |
| LSC01 | -13.602 | 127.550 | -0.11 | 0.915 | 0.0000 | 402 | 14600 | 0.028 | 0.000 | Forest - conifer | 44220.920 | Laesoe | Cattle |
| LSC01 | 0.106 | 1.002 | 0.11 | 0.916 | 1.1118 | 6154 | 14600 | 0.422 | 311.000 | Forest - mixed and broadleaved | 0.003 | Laesoe | Cattle |
| LSC01 | -11.608 | 1.000 | -11.61 | 0.000 | 0.0000 | 22 | 14600 | 0.002 | NA | Open - dry grassland | 1.000 | Laesoe | Cattle |
| LSC01 | -0.211 | 1.017 | -0.21 | 0.836 | 0.8096 | 5532 | 14600 | 0.379 | 203.589 | Open - dry heath and dunes | 0.005 | Laesoe | Cattle |
| LSC01 | 0.232 | 1.002 | 0.23 | 0.817 | 1.2607 | 788 | 14600 | 0.054 | 45.155 | Open wet | 0.022 | Laesoe | Cattle |
| LSC01 | -0.229 | 1.009 | -0.23 | 0.821 | 0.7954 | 1466 | 14600 | 0.100 | 53.000 | Scattered shrub and trees | 0.019 | Laesoe | Cattle |
| LSC01 | 0.572 | 1.026 | 0.56 | 0.577 | 1.7712 | 236 | 14600 | 0.016 | 19.000 | Wetland | 0.053 | Laesoe | Cattle |
| LSC02 | -0.711 | 1.080 | -0.66 | 0.510 | 0.4911 | 843 | 31301 | 0.027 | 6.000 | Forest - conifer | 0.167 | Laesoe | Cattle |
| LSC02 | 0.221 | 1.002 | 0.22 | 0.825 | 1.2474 | 13054 | 31301 | 0.417 | 236.000 | Forest - mixed and broadleaved | 0.004 | Laesoe | Cattle |
| LSC02 | -12.751 | 1.000 | -12.75 | 0.000 | 0.0000 | 69 | 31301 | 0.002 | NA | Open - dry grassland | 1.000 | Laesoe | Cattle |
| LSC02 | 1.494 | 1.005 | 1.49 | 0.137 | 4.4569 | 12004 | 31301 | 0.384 | 775.378 | Open - dry heath and dunes | 0.001 | Laesoe | Cattle |
| LSC02 | 1.702 | 1.001 | 1.70 | 0.089 | 5.4837 | 1672 | 31301 | 0.053 | 132.880 | Open wet | 0.008 | Laesoe | Cattle |
| LSC02 | 1.498 | 1.002 | 1.49 | 0.135 | 4.4728 | 3147 | 31301 | 0.101 | 204.000 | Scattered shrub and trees | 0.005 | Laesoe | Cattle |
| LSC02 | 2.021 | 1.009 | 2.00 | 0.045 | 7.5469 | 512 | 31301 | 0.016 | 56.000 | Wetland | 0.018 | Laesoe | Cattle |
| UNH01 | -1.445 | 0.140 | -10.34 | 0.000 | 0.2358 | 1704 | 103091 | 0.017 | 0.054 | Forest - conifer | 18.426 | Ulvshale_north | Horse |
| UNH01 | -2.022 | 0.062 | -32.71 | 0.000 | 0.1323 | 19957 | 103091 | 0.194 | 0.357 | Forest - mixed and broadleaved | 2.803 | Ulvshale_north | Horse |
| UNH01 | -10.524 | 0.032 | -331.96 | 0.000 | 0.0000 | 7403 | 103091 | 0.072 | NA | Open - dry grassland | 1.000 | Ulvshale_north | Horse |
| UNH01 | -0.885 | 0.041 | -21.78 | 0.000 | 0.4126 | 27842 | 103091 | 0.270 | 1.552 | Open - dry heath and dunes | 0.644 | Ulvshale_north | Horse |
| UNH01 | -0.997 | 0.040 | -24.78 | 0.000 | 0.3692 | 32953 | 103091 | 0.320 | 1.643 | Open wet | 0.609 | Ulvshale_north | Horse |
| UNH01 | -0.690 | 0.059 | -11.66 | 0.000 | 0.5014 | 5936 | 103091 | 0.058 | 0.402 | Scattered shrub and trees | 2.488 | Ulvshale_north | Horse |
| UNH01 | -2.506 | 0.279 | -8.99 | 0.000 | 0.0816 | 1185 | 103091 | 0.011 | 0.013 | Shrub dense | 76.538 | Ulvshale_north | Horse |
| UNH01 | -1.648 | 0.086 | -19.25 | 0.000 | 0.1924 | 6111 | 103091 | 0.059 | 0.159 | Wetland | 6.297 | Ulvshale_north | Horse |
| USC01 | -1.006 | 0.103 | -9.74 | 0.000 | 0.3657 | 3460 | 142456 | 0.024 | 0.172 | Forest - mixed and broadleaved | 5.800 | Ulvshale_south | Cattle |
| USC01 | -10.960 | 0.040 | -276.82 | 0.000 | 0.0000 | 7338 | 142456 | 0.052 | NA | Open - dry grassland | 1.000 | Ulvshale_south | Cattle |
| USC01 | -1.330 | 0.062 | -21.62 | 0.000 | 0.2645 | 19614 | 142456 | 0.138 | 0.707 | Open - dry heath and dunes | 1.415 | Ulvshale_south | Cattle |
| USC01 | -0.472 | 0.042 | -11.25 | 0.000 | 0.6240 | 96562 | 142456 | 0.678 | 8.212 | Open wet | 0.122 | Ulvshale_south | Cattle |
| USC01 | -0.643 | 0.071 | -9.02 | 0.000 | 0.5255 | 6216 | 142456 | 0.044 | 0.445 | Scattered shrub and trees | 2.246 | Ulvshale_south | Cattle |
| USC01 | -2.589 | 0.171 | -15.11 | 0.000 | 0.0751 | 5511 | 142456 | 0.039 | 0.056 | Shrub dense | 17.722 | Ulvshale_south | Cattle |
| USC01 | 0.089 | 0.066 | 1.35 | 0.176 | 1.0935 | 3755 | 142456 | 0.026 | 0.560 | Wetland | 1.787 | Ulvshale_south | Cattle |
| USC03 | 10.687 | 90.990 | 0.12 | 0.907 | 43785 | 406 | 15747 | 0.026 | 22502.291 | Forest - mixed and broadleaved | 0.000 | Ulvshale_south | Cattle |
| USC03 | -25.211 | 90.984 | -0.28 | 0.782 | 0.0000 | 790 | 15747 | 0.050 | NA | Open - dry grassland | 1.000 | Ulvshale_south | Cattle |
| USC03 | 14.561 | 90.984 | 0.16 | 0.873 | 2106881 | 2160 | 15747 | 0.137 | 5760586 | Open - dry heath and dunes | 0.000 | Ulvshale_south | Cattle |
| USC03 | 13.357 | 90.984 | 0.15 | 0.883 | 631924 | 10718 | 15747 | 0.681 | 8573372 | Open wet | 0.000 | Ulvshale_south | Cattle |
| USC03 | 14.856 | 90.984 | 0.16 | 0.870 | 2830533 | 672 | 15747 | 0.043 | 2407745 | Scattered shrub and trees | 0.000 | Ulvshale_south | Cattle |
| USC03 | 13.452 | 90.984 | 0.15 | 0.882 | 695351 | 588 | 15747 | 0.037 | 517552 | Shrub dense | 0.000 | Ulvshale_south | Cattle |
| USC03 | 13.614 | 90.985 | 0.15 | 0.881 | 817819 | 413 | 15747 | 0.026 | 427543 | Wetland | 0.000 | Ulvshale_south | Cattle |
| USH01 | -0.480 | 0.083 | -5.79 | 0.000 | 0.6185 | 3644 | 151448 | 0.024 | 0.293 | Forest - mixed and broadleaved | 3.410 | Ulvshale_south | Horse |
| USH01 | -11.001 | 0.039 | -278.53 | 0.000 | 0.0000 | 7685 | 151448 | 0.051 | NA | Open - dry grassland | 1.000 | Ulvshale_south | Horse |
| USH01 | -1.475 | 0.064 | -23.17 | 0.000 | 0.2288 | 21013 | 151448 | 0.139 | 0.626 | Open - dry heath and dunes | 1.599 | Ulvshale_south | Horse |
| USH01 | -0.404 | 0.042 | -9.70 | 0.000 | 0.6677 | 102710 | 151448 | 0.678 | 8.924 | Open wet | 0.112 | Ulvshale_south | Horse |
| USH01 | -0.806 | 0.075 | -10.68 | 0.000 | 0.4468 | 6493 | 151448 | 0.043 | 0.378 | Scattered shrub and trees | 2.649 | Ulvshale_south | Horse |
| USH01 | -2.303 | 0.148 | -15.54 | 0.000 | 0.1000 | 5877 | 151448 | 0.039 | 0.076 | Shrub dense | 13.082 | Ulvshale_south | Horse |
| USH01 | -0.039 | 0.068 | -0.57 | 0.569 | 0.9619 | 4026 | 151448 | 0.027 | 0.504 | Wetland | 1.985 | Ulvshale_south | Horse |
| AAC01 | -1.499 | 0.040 | -37.06 | 0.000 | 0.2234 | 46340 | 86460 | 0.536 | 1.380 | Forest - conifer | 0.724 | Aal | Cattle |
| AAC01 | -0.797 | 0.043 | -18.61 | 0.000 | 0.4508 | 17865 | 86460 | 0.207 | 1.074 | Forest - mixed and broadleaved | 0.931 | Aal | Cattle |
| AAC01 | -10.479 | 0.031 | -340.21 | 0.000 | 0.0000 | 7499 | 86460 | 0.087 | NA | Open - dry grassland | 1.000 | Aal | Cattle |
| AAC01 | -0.014 | 0.106 | -0.13 | 0.894 | 0.9859 | 700 | 86460 | 0.008 | 0.092 | Open - dry heath and dunes | 10.866 | Aal | Cattle |
| AAC01 | -1.521 | 0.064 | -23.63 | 0.000 | 0.2185 | 10190 | 86460 | 0.118 | 0.297 | Open wet | 3.367 | Aal | Cattle |
| AAC01 | -0.475 | 0.076 | -6.26 | 0.000 | 0.6221 | 2379 | 86460 | 0.028 | 0.197 | Scattered shrub and trees | 5.067 | Aal | Cattle |
| AAC01 | -1.093 | 0.269 | -4.06 | 0.000 | 0.3354 | 297 | 86460 | 0.003 | 0.013 | Shrub dense | 75.286 | Aal | Cattle |
| AAC01 | -1.208 | 0.145 | -8.34 | 0.000 | 0.2989 | 1190 | 86460 | 0.014 | 0.047 | Wetland | 21.080 | Aal | Cattle |
| AAC02 | -2.170 | 0.033 | -65.51 | 0.000 | 0.1142 | 61432 | 114560 | 0.536 | 0.714 | Forest - conifer | 1.401 | Aal | Cattle |
| AAC02 | -1.265 | 0.034 | -37.48 | 0.000 | 0.2823 | 23316 | 114560 | 0.204 | 0.670 | Forest - mixed and broadleaved | 1.493 | Aal | Cattle |
| AAC02 | -10.019 | 0.021 | -468.75 | 0.000 | 0.0000 | 9828 | 114560 | 0.086 | NA | Open - dry grassland | 1.000 | Aal | Cattle |
| AAC02 | -0.578 | 0.092 | -6.28 | 0.000 | 0.5612 | 1000 | 114560 | 0.009 | 0.057 | Open - dry heath and dunes | 17.512 | Aal | Cattle |
| AAC02 | -2.964 | 0.083 | -35.92 | 0.000 | 0.0516 | 13663 | 114560 | 0.119 | 0.072 | Open wet | 13.943 | Aal | Cattle |
| AAC02 | -1.412 | 0.078 | -18.16 | 0.000 | 0.2437 | 3298 | 114560 | 0.029 | 0.082 | Scattered shrub and trees | 12.229 | Aal | Cattle |
| AAC02 | -2.259 | 0.317 | -7.13 | 0.000 | 0.1044 | 430 | 114560 | 0.004 | 0.005 | Shrub dense | 218.900 | Aal | Cattle |
| AAC02 | -2.208 | 0.162 | -13.67 | 0.000 | 0.1099 | 1593 | 114560 | 0.014 | 0.018 | Wetland | 56.128 | Aal | Cattle |


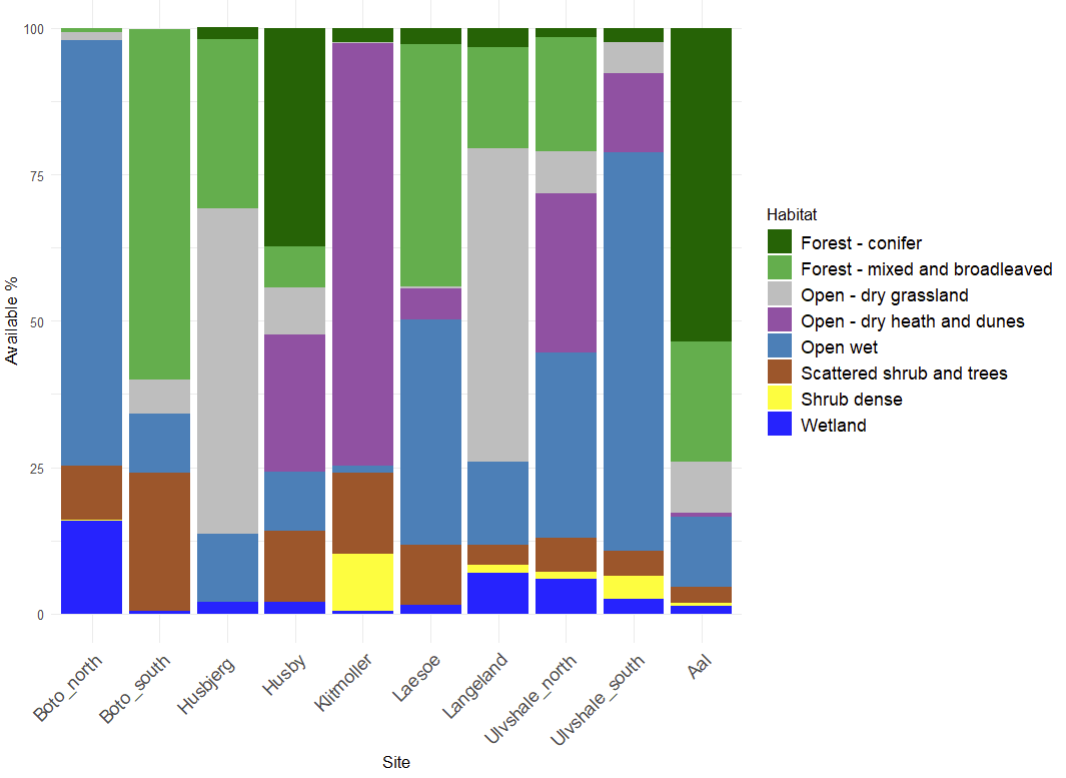


Figure S8 Habitat distribution (% cover) at each study site. Habitat cover varied between sites, with relatively open sites like Langland and Ulvshale North and South, while Boto South and Aal have large cover of closed woodland habitats.


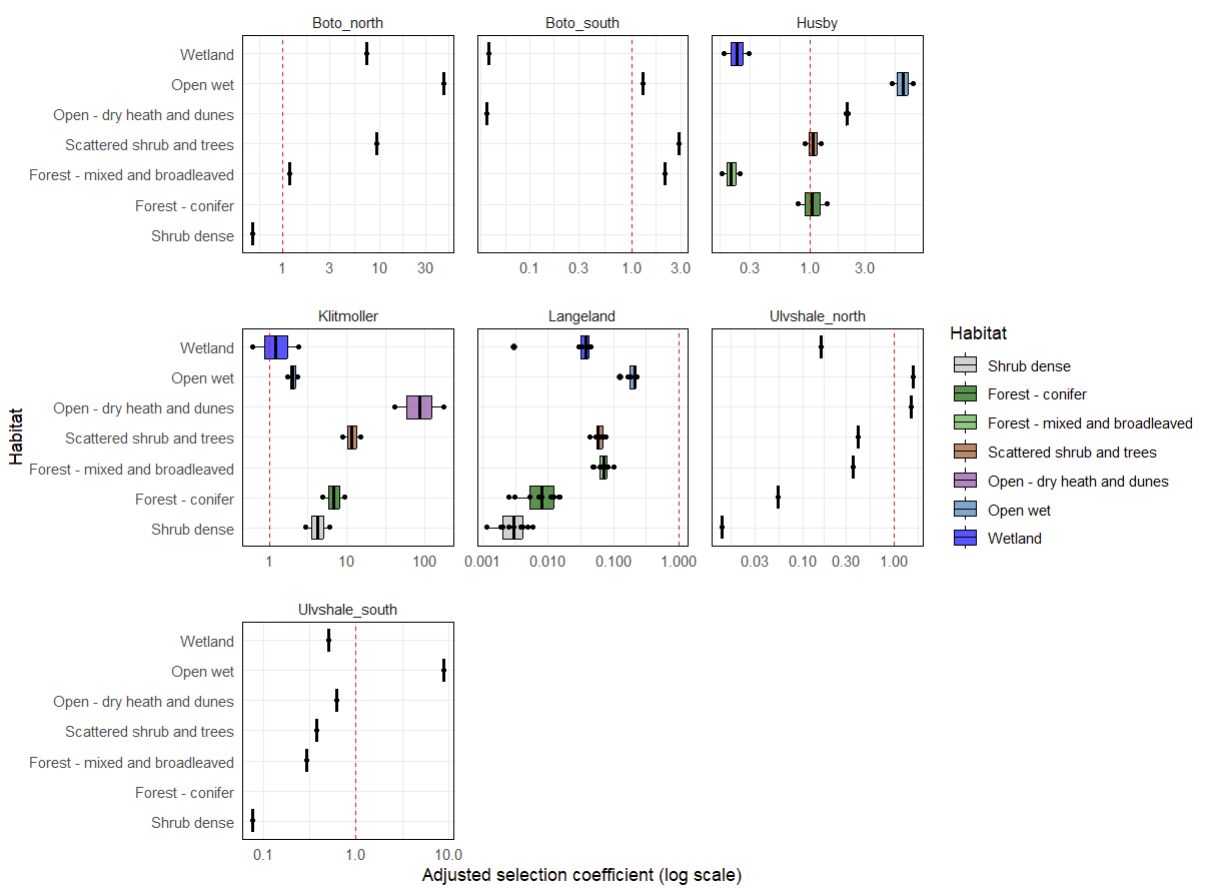


Figure S9 Adjusted selection coefficient for horses at all sites. Klitmøller (n= 2), Langeland (n=8), Husby (n= 2), the coefficients for individual animals at sites demonstrate that animals move in herds and selection in not individual. The study was based on GPS location data from seven study sites with horses and analysed using a resource selection function.


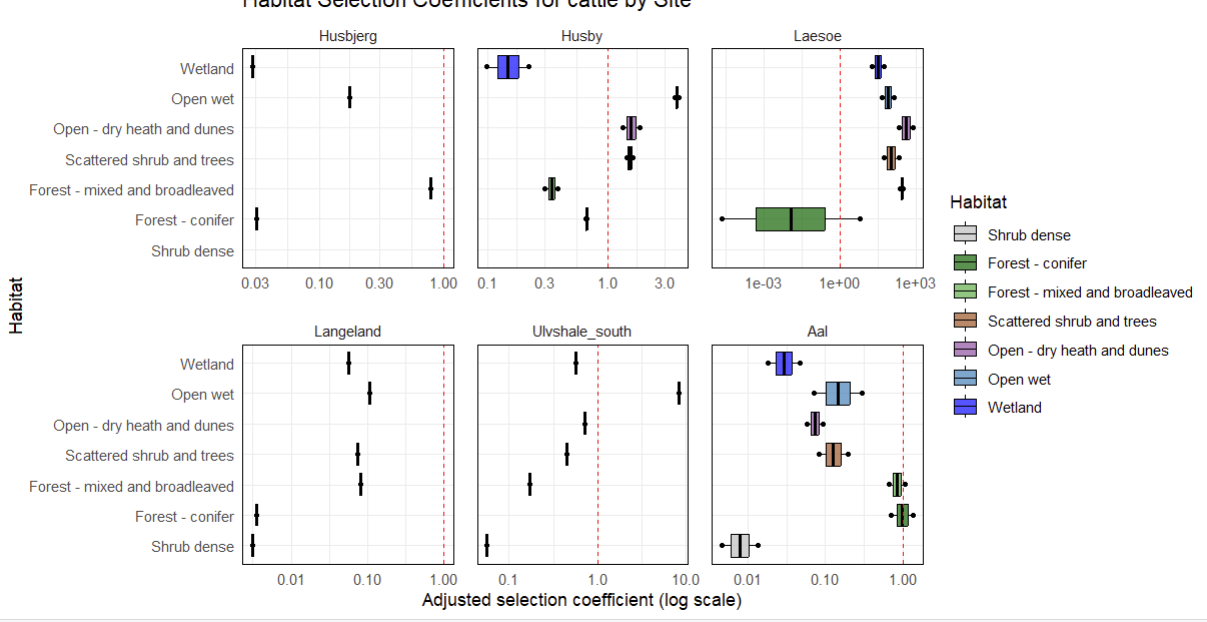


Figure S10 Adjusted selection coefficients for cattle at all six sites, Husby (n=2), Aal (n=2), Læsø (n=2). The coefficients for individual animals at sites demonstrate that individuals in same site have similar habitat use, apart from cattle at Læsø showing different use of conifer. The study was based on GPS location data and analysed using a resource selection function. Data from six study sites across Denmark with cattle.

Table S11 Distribution of observed behaviours in semi-feral horse and cattle

|  | Cattle | | Horse | |
| --- | --- | --- | --- | --- |
| Behaviour | N | % | N | % |
| Browsing | 3 | 0 | 17 | 1 |
| Drinking | 1 | 0 | 9 | 0 |
| Grazing | 362 | 45 | 1356 | 73 |
| Other | 8 | 1 | 26 | 1 |
| Resting | 279 | 35 | 54 | 3 |
| Rubbing | 13 | 2 | 25 | 1 |
| Standing | 121 | 15 | 274 | 15 |
| Walking | 19 | 2 | 101 | 5 |
| Wallowing | 1 | 0 | 0 | 0 |
| Lactating | 0 | 0 | 2 | 0 |
| Total | 807 | 100 | 1864 | 100 |
